# Evolution of casein kinase 1 and functional analysis of new *doubletime* mutants in *Drosophila*

**DOI:** 10.3389/fphys.2022.1062632

**Published:** 2022-12-14

**Authors:** Nirav Thakkar, Astrid Giesecke, Olga Bazalova, Jan Martinek, Vlastimil Smykal, Ralf Stanewsky, David Dolezel

**Affiliations:** ^1^ Biology Center of the Academy of Sciences of the Czech Republic, Institute of Entomology, Ceske Budejovice, Czechia; ^2^ Faculty of Science, University of South Bohemia, Ceske Budejovice, Czechia; ^3^ Institute of Neuro- and Behavioral Biology, Westfälische Wilhelms University, Münster, Germany

**Keywords:** casein kinase 1, doubletime, evolution, circadian clock, temperature compensation, bride of doubletime

## Abstract

Circadian clocks are timing devices that rhythmically adjust organism’s behavior, physiology, and metabolism to the 24-h day-night cycle. Eukaryotic circadian clocks rely on several interlocked transcription-translation feedback loops, where protein stability is the key part of the delay between transcription and the appearance of the mature proteins within the feedback loops. In bilaterian animals, including mammals and insects, the circadian clock depends on a homologous set of proteins. Despite mostly conserved clock components among the fruit fly *Drosophila* and mammals, several lineage-specific differences exist. Here we have systematically explored the evolution and sequence variability of insect DBT proteins and their vertebrate homologs casein kinase 1 delta (CKIδ) and epsilon (CKIε), dated the origin and separation of CKIδ from CKIε, and identified at least three additional independent duplications of the CKIδ/ε gene in *Petromyzon*, *Danio*, and *Xenopus*. We determined conserved regions in DBT specific to Diptera, and functionally tested a subset of those in *D. melanogaster*. Replacement of Lysine K224 with acidic residues strongly impacts the free-running period even in heterozygous flies, whereas homozygous mutants are not viable. K224D mutants have a temperature compensation defect with longer free-running periods at higher temperatures, which is exactly the opposite trend of what was reported for corresponding mammalian mutants. All DBTs of dipteran insects contain the NKRQK motif at positions 220–224. The occurrence of this motif perfectly correlates with the presence of BRIDE OF DOUBLETIME, BDBT, in Diptera. BDBT is a non-canonical FK506-binding protein that physically interacts with *Drosophila* DBT. The phylogeny of FK506-binding proteins suggests that BDBT is either absent or highly modified in non-dipteran insects. In addition to *in silico* analysis of DBT/CKIδ/ε evolution and diversity, we have identified four novel casein kinase 1 genes specific to the *Drosophila* genus*.*

## Introduction

To cope with and anticipate daily environmental changes, organisms have evolved circadian clocks. These genetically determined time-measuring devices “tick” with a free-running period (τ) close to 24 h (Dunlap, 1999). The circadian clock runs with almost the same τ within the physiological temperature range; this phenomenon, known as *temperature compensation*, seemingly contradicts the basic principles of biochemical reactions (Arrhenius, 1889). At the molecular level, circadian clocks in eukaryotes comprise interlocked negative transcription-translation feedback loops (TTFL; Dunlap, 1999). The positive regulators are transcription factors driving the expression of mRNAs encoding the negative regulators. Once the negative regulators are present in the nucleus, they inhibit their own expression by suppressing the activity of the positive regulator(s). Importantly, the mere transcription-translation process would be much faster than the required 24-h cycle, thus, additional steps delaying the entire process must be involved. Firstly, the negative regulator proteins are initially destabilized, which delays their accumulation. In addition, the translocation of the negative regulators to the cell nucleus might require dimerization with a partner protein, and often larger complexes are formed (Aryal et al., 2017). At a biochemical level, both positive and negative regulators undergo various posttranslational modifications, of which protein phosphorylation is the most prominent. In the end, well-timed depletion of the negative regulators is key for the start of the next cycle and contributes to the resulting τ.

### *Drosophila* and mammalian clock

The circadian clock of mammals and the fruit fly *Drosophila melanogaster* relies on homologous components. The positive regulators CLOCK and BMAL/CYCLE belong to the basic helix-loop-helix (bHLH) PER-ARNT-SIM (PAS) transcription factors (King et al., 1997; Darlington et al., 1998; Hogenesch et al., 1998; Rutila et al., 1998). PERIOD (PER), which also belongs to the PAS protein family, is a negative regulator shared among vertebrates and insects (Hardin et al., 1990; Zylka et al., 1998).

In *D. melanogaster*, PER interacts with *Drosophila*-type TIMELESS protein (dTIM) (despite the general conservation of the molecular mechanisms and genetic components among various vertebrates and insects, some important differences exist. Furthermore, the gene/PROTEIN names vary in the literature as they were historically evolving. Here, we use the prefix m-for the so-called mammalian type and the prefix d-for the *Drosophila*-type proteins. See the supplementary text for more detailed notes on circadian clock gene terminology).

PER:dTIM dimerization in the cytoplasm is necessary for subsequent nuclear localization of PER and dTIM (Saez and Young, 1996; Meyer et al., 2006). dTIM is an essential component of the fruit fly circadian clock, because *tim* null mutations result in complete arrhythmicity (Sehgal et al., 1994), whereas missense mutations affect τ (Rothenfluh et al., 2000; Wulbeck et al., 2005), and certain *d-tim* mutations affect the temperature compensation of the circadian clock (Matsumoto et al., 1999; Singh et al., 2019). Furthermore, dTIM is a key component of the light-mediated synchronization in *Drosophila* (Hunter-Ensor et al., 1996; Myers et al., 1996; Zeng et al., 1996), which involves light-dependent interaction with *Drosophila*-type CRYPTOCHROME (dCRY) (Ceriani et al., 1999; Peschel et al., 2009). dCRY serves as a deep brain circadian photoreceptor (Emery et al., 2000) with no impact on the behavioral rhythmicity in constant-dark conditions (DD) at ambient temperature (Stanewsky et al., 1998), although *d-cry* depletion reduced rhythmicity at 18°C (Dolezelova et al., 2007). Interestingly, *d-cry* mutations abolish transcriptional oscillations in peripheral clocks, which allowed the identification of this mutant in a luciferase reporter-based screen (Stanewsky et al., 1998). In mice, mammalian-type CRYPTOCHROME (mCRY) is present as two paralogous and closely related proteins, that are essential for (light-independent) clock function, while dimerizing with one of the three mammalian PER proteins (Zylka et al., 1998; Kume et al., 1999; Putker et al., 2021).

An important feature of the negative TTFL is the temporal regulation of subcellular localization of participating proteins. In addition to nuclear localization signals (NLS), some circadian clock proteins also contain nuclear export signals (NES) (Saez and Young, 1996; Vielhaber et al., 2001; Ashmore et al., 2003; Yildiz et al., 2005; Hara et al., 2011; Saez et al., 2011; Jang et al., 2015; Singh et al., 2019; Giesecke et al., 2021). Thus, the resulting nuclear import/export strongly affects the suppression potential of the negative feedback loop and thereby τ. The stability and subcellular localization of the negative complex, such as PER and dTIM, is regulated by posttranslational modifications (Li et al., 2019; Crosby and Partch, 2020), including phosphorylation and dephosphorylation by several kinases and phosphatases (Sathyanarayanan et al., 2004; Leloup 2009; Reischl and Kramer, 2011; Agrawal and Hardin, 2016; Narasimamurthy and Virshup., 2021). One of the most explored circadian clock kinases is DBT which was first identified as a clock component in a Drosophila screen when the short- (DBT^S^) and long- (DBT^L^) free-running period mutants were identified (Price et al., 1998; Kloss et al., 1998, Figure 8). However, as it turned out later, *dbt* is also known as *discs overgrown*, a gene which had been discovered for its role during development (Jursnich et al., 1990; Zilian et al., 1999). Mammalian homologs of DBT are CKIδ/ε, which were shown to be essential for the clock in the hamster, human, and mice (Lowrey et al., 2000; Xu et al., 2005; Meng et al., 2008). The interaction between this kinase and PER is remarkably stable (Kloss et al., 2001; Lee et al., 2001; Aryal et al., 2017). Overexpression of either DBT^L^ or DBT^S^ variants in *Drosophila* resulted in the same τ as was produced by the corresponding alleles of the endogenous gene (Muskus et al., 2007), whereas *in vitro* studies using non-physiological substrates implied, surprisingly, that both mutants have reduced kinase activity (Kivimaë et al., 2008; Venkatesan et al., 2019). Furthermore, unlike most enzymes, CKIδ/ε activity is temperature insensitive (Isojima et al., 2009), but paradoxically, the hamster CK1ε^tau^ mutant is a temperature compensation mutant (Tosini and Menaker, 1998). The conundrum started to unravel in the context of the PER phosphorylation pattern elicited upon the action of multi-kinase hierarchical activities identified in several model organisms (Xu et al., 2007; Ko et al., 2010; Chiu et al., 2011; Lam et al., 2018). The current phosphoswitch model involves two competing phosphorylation sites on mouse (*Mus musculus*) PER2, the phosphodegron and the FASP (familial advanced sleep phase, Toh et al., 2001) sites, which regulate PER2 stability in opposing ways (Zhou et al., 2015; Masuda et al., 2020). Thus, the temperature-sensitive phosphoswitch slows down PER2 degradation at higher temperatures, resulting in a global temperature-compensated system. Somewhat similar, multiple phospho-clusters are detected on *Drosophila* PER, which cumulatively contribute to PER stability and transcriptional repressor activity (Chiu et al., 2008; Kivimaë et al., 2008; Garbe et al., 2013; Top et al., 2018). Therefore, the phosphoswitch mechanism might be conserved across species, even though the details differ, as a phosphodegron with functionally heterogeneous sites was recently reported for *Drosophila* (Joshi et al., 2022).

Both in mammals and *Drosophila*, the PER phosphorylation pattern is defined by the synergistic action of multiple kinases (see the text above), phosphatases (Sathyanarayanan et al., 2004; Fang et al., 2007; Reischl and Kramer, 2011), and some additional post-translational modifications, such as O-GlcNAcylation and acetylation (Kaasik et al., 2013; Li et al., 2019). The PER phosphorylation dynamics is regulated by yet another level of complexity, as is indicated by the distinct capacity of CKIδ splice isoforms. CKIδ1 and CKIε (both similar in the last 16 amino acids of their carboxy-terminal tails, here abbreviated as “C-terminal tails”) are more active in priming kinase activity at the FASP site, whereas CKIδ2 is more potent in priming the degron site (Fustin et al., 2018; Narasimamurthy et al., 2018). The CKIδ/ε C-terminal tail autophosphorylation inhibits its kinase activity (Graves and Roach, 1995; for review see Narasimamurthy and Virshup, 2021). As was shown for *Drosophila* DBT, the C-terminal tail stabilizes interactions between the kinase and the substrate, while the C-terminal tail autophosphorylation inhibits substrate binding (Dahlberg et al., 2009; Fan et al., 2015). Furthermore, two residues on the DBT kinase domain influence its affinity to PER (Dahlberg et al., 2009). However, no splicing isoforms of DBT exist in *Drosophila* as *dbt* is an intronless gene in this species.

The temperature-independent activity of CK1δ/ε was connected to sequence motifs close to the active site of the kinase, where Lysine 224 was identified as key for the temperature-compensated primed phosphorylation (Shinohara et al., 2017). Importantly, the K224D mutation in CK1δ shortens τ and affects temperature compensation in the mammalian system *in vitro*. Notably, the corresponding region of *Drosophila* DBT was systematically explored by Venkatesan et al. (2019) who identified a second NLS in positions 220–224. This region in DBT is further important for its interaction with BRIDE of DOUBLETIME (BDBT) (Venkatesan et al., 2015), a non-canonical FK506-binding protein with tetratricopeptide repeats that might promote the assembly of larger protein complexes (Fan et al., 2013).

Although the circadian clock is in general conserved among bilaterian species, some notable variations in the PER/dTIM/dCRY/mCRY feedback exist with some functional implications (Kotwica-Rolinska et al., 2022a). Therefore, we decided to explore and define the variability in insect DBT proteins. As a reference, we analyzed deuterostomian homologs of DBT and dated the origin and separation of CKIδ from CKIε. Furthermore, we have identified four novel casein kinase I genes specific to the *Drosophila* genus*.* We identified conserved regions in DBT specific to Diptera, functionally tested some of them in *D. melanogaster*, and analyzed their impact on temperature compensation of the circadian clock.

## Materials and methods

Recent progress in genome and transcriptome sequencing (Misof et al., 2014; Johnson et al., 2018; Kawahara et al., 2019; McKenna et al., 2019; Wipfler et al., 2019) allowed us to systematically explore casein kinases and FK506-binding proteins across all major insect orders. In essence, we applied an approach similar to that of Smykal et al. (2020), when multiple rounds of Basic Local Alignment Search Tool (BLAST) searches followed by fast phylogenetic analyses were conducted to retrieve evolutionary informative sequences from the genomes and transcriptomes of all major insect lineages. Although a reasonable collection of sequences could be retrieved from the protein database using BLASTP algorithm, more detailed and taxon-focused TBLASTN searches (search in translated nucleotide databases using a protein query) were used to explore transcriptome shotgun assemblies (TSAs). Multiple query sequences were tested in all searches described above (fruit fly *Drosophila melanogaster* DBT, firebrat *Thermobia domestica* DBT, and house mouse *Mus musculus* CKIε/CKIδ). For well-annotated genomes (zebrafish *Danio rerio*, African clawed frog *Xenopus laevis*, *M. musculus*, human *Homo sapiens*, etc.), all protein variants were retrieved directly from gene models. To retrieve non-DBT/CKIε/CKIδ kinases, multiple rounds of BLASTP and TBLASTN were performed. To test whether *Drosophila*-specific CKI genes (CG9962, CG2577, CKIalpha-like I, and CKIalpha-like II) could be identified outside of *Drosophila*, TBLASTN was performed in TSA of all insects with the exclusion of the *Drosophila* genus (NCBI:txid7215). In addition, reciprocal BLAST searches were performed when the identified sequence served as a query in the next rounds of BLASTs. Additional *dbt* sequences were obtained by PCR and 3′RACE from the housefly *Musca domestica* (Bazalova and Dolezel, 2017) and *Chymomyza costata* (Kobelkova et al., 2010), with support from Illumina-based transcriptome (Poupardin et al., 2015). See Supplementary Tables S1, S2 for accession numbers.

To reconstruct the evolution of BDBT, all FK506-binding protein homologs were retrieved from *D. melanogaster*, the monarch butterfly *Danaus plexippus*, the red flour beetle *Tribolium castaneum*, the brown marmorated stink bug *Halyomorpha halys*, and *M. musculus*. Then, multiple rounds of order- and species-specific searches in insects were employed and fast phylogenetic analyses performed. First, proteins were aligned using the algorithm MAFFT E-INS-i in Geneious 11 (Biomatters). Then, a FAST tree algorithm in Geneious 11 (Biomatters) was used to infer preliminary trees and identify duplicates. For detailed analyses, protein sequences were aligned using MAFFT algorithm with the E-INS-i multiple alignment method and the BLOSUM80 scoring matrix, and the trees were inferred using RAxML maximum likelihood GAMMA-based model and the bootstrap values calculated from 100 replicates (both as a package of Geneious 11 software, Biomatters). The datasets consisted of 239 sequences used for CKI evolution in Figure 2, whereas 31 sequences were used for vertebrate-specific duplication analyses (Figure 4), and 280 sequences were used for BDBT/FK506-binding proteins (Figure 5).

### *Pyrrhocoris apterus* Oxford nanopore technology mRNA sequencing

Details of Oxford Nanopore Technology (ONT) transcriptome sequencing will be described elsewhere. Briefly, *P. apterus* brains and other tissues were dissected and poly A+ mRNA was isolated using Dynabeads mRNA DIRECT Kit (Life Technologies) according to the manufacturer’s instructions and 100 ng of the polyA+ mRNA was then reverse-transcribed, turned to double-stranded DNA, and the sequencing adaptors were added using PCR-free Direct cDNA Sequencing kit (SQK-DCS109; Oxford Nanopore Technology) according to the manufacturer’s instructions. The library was immediately sequenced on a MinION device (Oxford Nanopore Technology). Base calling was performed after the run using Guppy 3.6.0 at a high-accuracy setting. Obtained tissue-specific transcriptomes were used in exhaustive searches using *P. apterus dbt* mRNA sequence as a query. All *dbt* transcripts were retrieved, manually inspected, and mapped to the in-house *P. apterus* genome (hybrid assembly of Illumina and ONT data, which will also be published separately), and a *dbt* gene model was built. All *dbt* transcripts were sequentially mapped to four defined individual *dbt* isoforms and only reads unequivocally distinguishing specific *dbt* isoform (protein)-coding sequences were counted.

### Phosphorylation prediction

The putative phosphorylation sites were predicted *in silico* using NetPhos 3.1 server at http://www.cbs.dtu.dk/services/NetPhos/ and scores higher than 0.5 were plotted in alignments.

### Gene editing inducing non-homologous-end-joining (NHEJ) mutants

The target site was designed to induce a double-strand strand brake in the C-terminal tail coding part of *dbt* gene. Two gRNA sequences (PAM, which is not part of the gRNA, is shown in square brackets) targeting GCG​ATG​CTG​GGC​GGC​AAT​GG[AGG] and GTC​GGC​CTT​CGA​TAC​GGA​TG[CGG], respectively, were prepared from custom-synthesized oligonucleotides and cloned into pBFv-U6.2 (Kondo and Ueda, 2013) obtained from fly stocks of National Institute of Genetics, Japan (NIG-FLY). Plasmids were injected into *y* ^*1*^ *v* ^*1*^ *P{nos-phiC31\int.NLS}X; attP40 (II)* (NIG-FLY#: TBX-0002) flies with docking site on the second chromosome, transformants identified by eye color rescue, and balanced by *y* ^*2*^ *cho* ^*2*^ *v* ^*1*^ */Y* ^*hs-hid*^ *; Sp/CyO* (NIG-FLY#: TBX-0008).

Flies expressing Cas9 specifically in germ cells (nos-Cas9) from the second chromosome insertion (NIG-FLY#: CAS-0001; *y* ^*2*^ *cho* ^*2*^ *v* ^*1*^ *; attP40{nos-Cas9}/CyO*) were crossed with U6gRNA-encoded transgenic strains (also located on the second chromosome). Resulting F1 offspring thus expressed both gRNA and CAS9 on second chromosomes, which potentially targeted the *dbt* gene located on the third chromosome and induce insertions and deletions as a result of the non-homologous-end-joining (NHEJ) mechanism. The resulting F1 offspring were crossed to *y* ^*2*^ *cho* ^*2*^ *v* ^*1*^ *; Pr Dr/TM6C, Sb Tb* (NIG-FLY#: TBX-0010) to balance the modified third chromosomes with TM6C. Individual F1 flies were used in heteroduplex mobility shift assay (Kotwica-Rolinska et al., 2019) to identify flies with the highest degree of mosaicism in the targeted *dbt* locus, thus, the crosses with the highest frequency of NHEJ-induced mutants were identified. From these selected crosses, F2 males and females with third chromosome balancer were individually crossed back to *y* ^*2*^ *cho* ^*2*^ *v* ^*1*^ *; Pr Dr/TM6C, Sb Tb* flies (NIG-FLY#: TBX-0010) to establish lines with identically modified third chromosomes. Mutated region was identified by polymerase chain reaction (PCR) and sequencing.

### Gene editing inducing homology directed repair (HDR)—gRNA design

Target gRNA sites were selected so that Cas9-mediated cleavage was directed to a target locus of 100 bp upstream and downstream of the *dbt* K244 site. To avoid off-target cleavage optimal target sites were identified using CRISPR target finder (http://flycrispr.molbio.wisc.edu/tools). One gRNA target was chosen that was close to the target locus. Complementary target site oligos also contained a 5′ guanine for transcription from the U6 promoter and a 3 bp overhang compatible with BbsI sites. Oligos were annealed using standard primer annealing reactions and cloned into BbsI linearized pCFD3 plasmid (Port et al., 2014) *via* T4 DNA ligation.

Donor plasmids that contain the desired *dbt* mutations and all elements necessary for homologous recombination were constructed in 3 subsequent cloning steps. In each round of cloning the 1.5 kb 5′ homology arm and the 1.5 kb 3′ homology arm were individually PCR amplified from nos-Cas9 flies (Port et al., 2014) using outside primers dbtBMHRF and dbtBMHRR in combination with respective internal primers. Outside primers dbtBMHRF and dbtBMHRR contain a 15 bp overhang for In-Fusion cloning that is homologous to linearized vector ends. Inside primers have 5′ 15–20 bp extensions that are complementary to each other in addition to one defined mutation for each round of cloning. In the initial round of cloning a silent SalI site was introduced that can be used to screen for transformants. The two fragments (5′ homology arm and 3′ homology arm) were assembled into plasmid pBS-KS-attB1-2-PT-SA-SD-0-2xTY1-V5 (Addgene) that was linearized with XbaI and HindIII using In-Fusion cloning. In a second round of cloning the homology arms were amplified again using the pBS donor plasmid from the previous round as a template. Outside primers were as described above while the inside primers introduced either the K224D or the K224E mutation, respectively. In-Fusion cloning was used to assemble the fragments as described above. The resulting plasmid was then used in a final round of PCR to introduce PAM site mutations to avoid unwanted Cas9 cleavage within the donor plasmid. See Supplementary table S3 for a detailed list of all primers.

Donor plasmids containing the desired mutation along with gRNA plasmids were verified by sequence analysis and scaled up for injections using Qiagen plasmid midiprep. 6 µg of each plasmid were precipitated and eluted in injection buffer. gRNA construct and donor plasmids were mixed prior to injection and the mix was injected into freshly laid embryos of *nos-Cas9* flies (Port et al., 2014). Surviving adults were backcrossed in batch crosses to *y w*; +; *Dr/TM3* flies to balance 3rd chromosome modifications. Individual male and female flies from this cross were crossed again to *y w*; +; *Dr/TM3*. After letting the females lay eggs for 3–5 days, adult transformant flies were used for molecular screening.

### Molecular screening in HDR experiments

In general, a total of 95 flies for each mutation were screened using PCR in combination with restriction digests. A ∼800 bp target locus was amplified by PCR using genomic DNA from individual flies. 20 units of SalI were then added to half of the PCR reaction and incubated for 2 h at 37°C. The resulting products were analyzed on agarose gels. The remaining PCR product of samples that showed digested products of the correct size were then used for sequencing to verify the presence of the desired mutations.

### Locomotor activity recordings and analysis

No homozygous flies could be obtained for both K224 *dbt* mutations and both stocks are balanced over TM3. Thus, for behavior experiments, flies harboring the *dbt* mutations were crossed against *y w* controls and only flies without TM3 were tested. For *dbt* C-terminal tail mutants, homozygous flies were tested.

Two to four-day old males were loaded into glass tubes containing 5% sucrose in 2% agar and loaded into the DAM2 TriKinetics system (Waltham, MA) and locomotor activity was recorded as previously described (Pfeiffenberger et al., 2010). K224 mutant flies were exposed to 12 h Light: 12 h Dark regime (LD) for 3 days, followed by 5–7 days in constant darkness (DD) to assess their free-running periods at constant temperatures of 18°C, 25°C, or 29°C. Period length and their significance (RS values) were determined using autocorrelation and Chi-square periodogram analysis functions of the fly toolbox implemented in MATLAB (MathWorks) (Levine et al., 2002). Period values with associated RS values ≥1.5 were considered rhythmic (Levine et al., 2002).

Two to four-day old C-terminal tail mutant males were loaded into the DAM2 TriKinetics system as described above, exposed to LD for 5 days, followed by 10 days in DD to assess their free-running periods at constant temperatures of 17°C, 20°C, 25°C, or 28°C. To determine τ during the first 10 days in DD, Lombe-Scargle periodogram analysis was performed using ActogramJ (Schmid et al., 2011) and double-plotted actograms were eye inspected in parallel.

## Results

### *Drosophila* DBT diverges both from mammalian and from ancestral insect homologs

**FIGURE 1:**
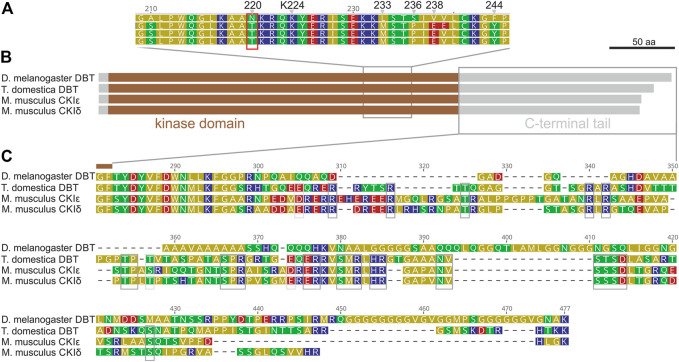
*Drosophila melanogaster* DBT differs from DBT of ancestral insect *Thermobia domestica* and both mouse (*M. musculus*) homologs, CKIε and CKIδ. **(A)** A detail of the kinase domain with highlighted conserved Lysine K224 and *Drosophila*-specific differences: N220, L233, S236, V238, and F244. **(B)** A schematic depiction of proteins with highlighted kinase domain, N- and C-terminal tails, and positions of detailed alignments shown in panels **(A,C)**. **(C)** Detail of the C-terminal tail, where grey boxes indicate residues conserved among CKIε, CKIδ, and *T. domestica* DBT.

Although mammalian CKIε, CKIδ, and *Drosophila* DBT are conserved components of the circadian clock, the mouse *CKIε* sequence did not rescue either the lethality or the rhythmicity of *dbt*-deficient *Drosophila* (Sekine et al., 2008). Therefore, to identify the key differences, we compared the protein sequences of mouse CKIε and CKIδ, *Drosophila melanogaster* DBT, and DBT of the most basal insect, the firebrat *Thermobia domestica* (Figure 1). All four proteins consist of the conserved casein kinase domain with a substantial C-terminal tail, whereas the N-terminal extension is minimal. However, a detailed inspection revealed two major differences. Within the kinase domain, the region 210–244 of *D. melanogaster* DBT differs from all three sequences. Notably, *D. melanogaster* DBT contains Asparagine (N) instead of Threonine (T) in position 220, which contrasts with DBT homologs from *Thermobia*, mouse, (Figure 1A), and more distant kinases. A recent study indicates that autophosphorylation of T220 influences substrate specificity (Cullati et al., 2022), thus the N at 220 prevents this posttranslational regulatory modification. Remarkable sequence divergence is observed in the C-terminal tail. Although the tail contains positions conserved even among both mouse CKI sequences and *T. domestica* DBT, surprisingly, the tail sequence is quite different in *Drosophila* with major insertions and deletions compared to the mammalian and firebrat sequences. These data indicate that during insect evolution DBT acquired substantial changes present in recent *D. melanogaster* DBT.

### Evolution of casein kinases in insects and deuterostomia

**FIGURE 2:**
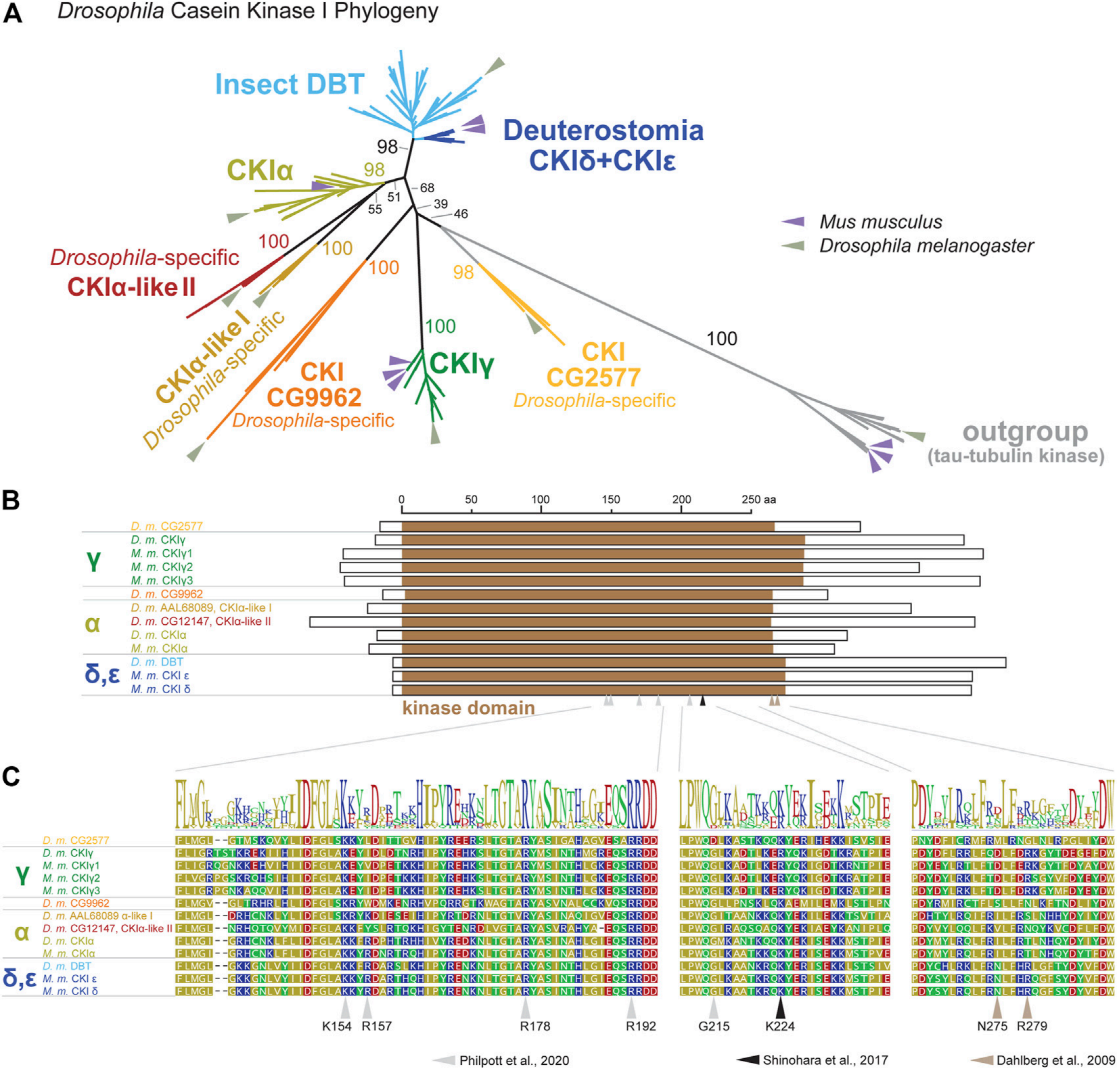
Phylogeny of bilaterian Casein kinase I (CKI) reveals seven clearly separated CKI-coding genes in fruit flies (*Drosophila* genus). **(A)** A tree illustrating relatedness among CKI proteins (values above branches indicate bootstrap support), in which well-separated clusters are color-coded. Insect DBTs (cerulean) branch together with deuterostomian sequences (cobalt blue) including CKIε and CKIδ from fish, amphibia, reptiles, and mammals. Besides well-established isoforms CKIα and CKIγ (the latter encoded by *gilgamesh* in *Drosophila*), four *Drosophila* genus-specific clusters were detected. Two of them branch at the base of CKIα and are therefore labeled as CKIα-like I (peanut) and CKIα-like II (cinnamon brown). Two additional clusters are separated from established CKI isoforms and are labeled according to the *D. melanogaster* nomenclature as CG9962 (orange) and CG2577 (apricot). Tau-tubulin kinases (a.k.a. *asator* in *D. melanogaster*) serve as an outgroup. Positions of the fruit fly *D. melanogaster* and the mouse *Mus musculus* proteins are highlighted by arrows in sage green and lavender, respectively. In established CKI terminology, the term isoform (α, γ, δ, ε) is used to refer to kinases encoded by distinct genes, although some of these genes might also encode different splice variants (for clarity, we use the term “splicing isoforms”). Multiple arrows in the mouse refer to gene multiplications, not to splicing isoforms. The phylogenetic analysis strongly supports the existence of the new groups of CKI genes in *Drosophila* and confirms already established groups. However, the relationship among CKI groups is sometimes poorly supported and the tree should not be interpreted as a focused analysis of CKI history. The tree was inferred using RAxML maximum likelihood of 239 protein sequences (final GAMMA-based score of the best tree -72993.763796) using Geneious 11 software (Biometters). Bootstrap support was calculated from 100 replications. See Supplementary Tables S1, S2 for accession numbers of analyzed sequences. **(B)** Schematic illustration of CKI proteins with highlighted kinase domain (brown), N- and C-terminal tails are shown as empty rectangles. **(C)** Details of protein alignment with highlighted residues that are important for the function of CKIδ (Shinohara et al., 2017; Philpott et al., 2020) and DBT (Dahlberg et al., 2009).

To be able to perform a comprehensive analysis of DBT/CKIε/CKIδ evolution, we first explored the phylogeny of casein kinases I in insects and used representative deuterostomian species as a reference. Tau-tubulin kinase served as an outgroup. CKI formed five distinct clusters (Figure 2A and Supplementary Figure S1): DBT/CKIε/CKIδ, CKIα, CKIγ, and two additional clusters not assigned to a specific CKI-type. These two clusters, provisionally labeled as CG9962 and CG2577, seem to be specific to the *Drosophila* genus, as no representative was found even in the dipteran genera *Musca* or *Ceratitis*. Similarly, two *Drosophila*-specific clusters are branching at the base of CKIα; thus, we use the provisional terms CKIα-like I and CKIα-like II (see Supplementary Table S1 for all non-DBT acc. numbers and Supplementary Table S2 for DBT, CKIε, and CKIδ acc. numbers). In line with the observed phylogenetic clustering of CKI, differences were identified in the N- and C-terminal tail lengths (Figure 2B) and in the sequence motifs within the kinase domain, including the activation loop (Philpott et al., 2020) and residues N275 and R279, which are responsible for enhanced substrate-specific binding to DBT (Dahlberg et al., 2009) (Figure 2C).

### Dbt, CKIε, and CKIδ genes in insects and deuterostomia

Having unambiguously identified CKI types, we performed a systematic audit of the DBT sequences across insects with three goals: explore possible patterns in the C-terminal tail variability, determine when the NKRQK motif arose, and identify whether we may correlate these changes in DBT with additional changes in the circadian clock setup.

**FIGURE 3:**
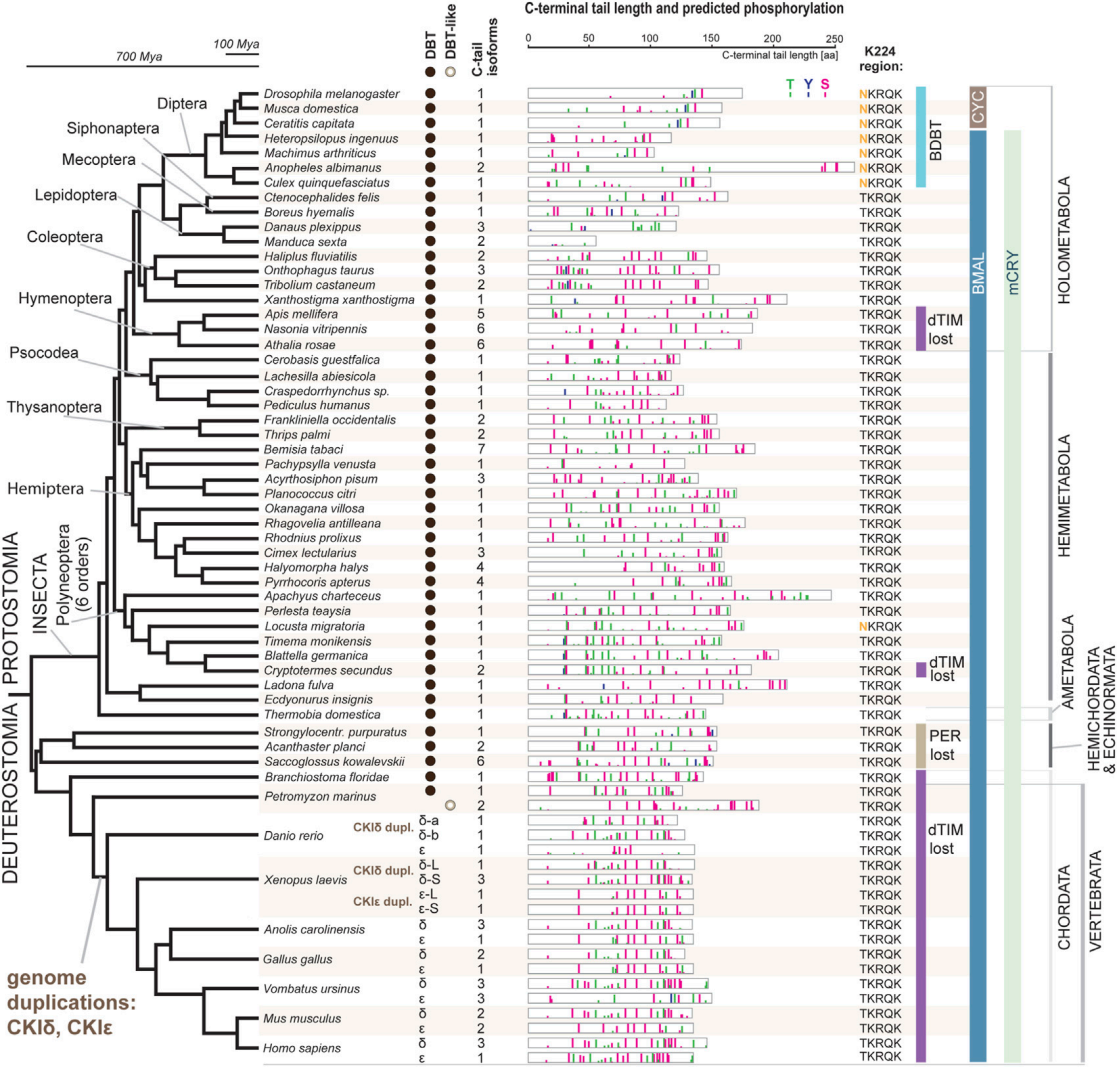
DBT and CKIδ/ε genes and proteins mapped on insect and deuterostomian phylogeny. The phylogenetic tree corresponds to a consensus of recent phylogenomic studies (Misof et al., 2014; Johnson et al., 2018; Kawahara et al., 2019; McKenna et al., 2019; Wipfler et al., 2019). Representative species are shown at the terminal nodes. The first column indicates the presence of DBT-coding genes (note a *Petromyzon*-specific gene duplication). Greek letters refer to the presence of CKIδ and CKIε; note two CKIδ paralogs in *Danio* and *Xenopus* and two CKIε paralogs in *Xenopus* (for details on CKIδ and CKIε phylogeny, see Figure 4). The second column indicates how many splicing isoforms affecting the C-terminal tail protein sequence were identified. The C-terminal tail of the longest isoform is depicted for each gene in each species (see Supplementary Figures S2–S7 for all isoforms and additional species). The color bars indicate *in silico* predicted phosphorylation patterns of threonine (T, green), tyrosine (Y, blue), and serine residues (S, pink). The bar’s height refers to the predicted score between 0.5–1.0. K224 region indicates whether NKRQK or TKRQK motif was found in the region corresponding to the 220–224 position within the catalytic domain of *D. melanogaster* DBT. Major changes in the circadian clock setup are depicted: presence of CRY mammalian (mCRY) type, loss of TIMELESS-drosophila type (dTIM), loss of PERIOD (PER) (Kotwica-Rolinska et al., 2022b), and transition of BMAL (activation domain is present) to CYC (activation domain lost) (Supplementary Figure S8). DBT-interacting protein paralogs BRIDE of DBT (BDBT) was found only in Diptera (for details and BDBT phylogeny see Figure 5).

**FIGURE 4:**
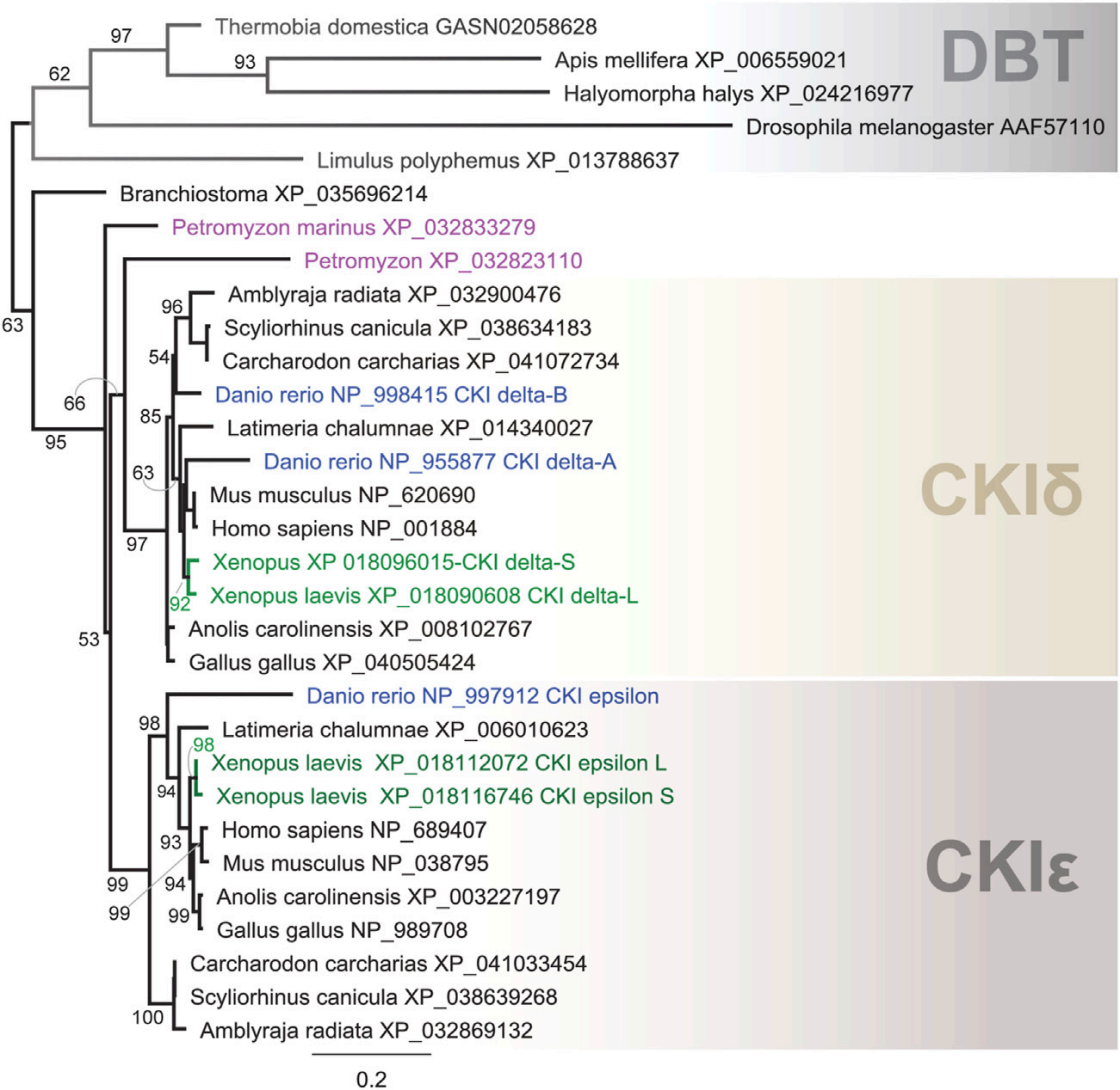
Phylogeny of DBT, CKIδ, and CKIε indicates several gene duplications in vertebrates. In the lancelet *Branchiostoma*, only one CKI gene was found to precede the δ- and ε-isoforms. The major duplication that gave rise to the δ and ε isoforms dates back to the ancestor of Gnatostomata (vertebrates with jaws), while two CKI genes in the sea lamprey *Petromyzon marinus* resulted from *Petromyzon*-specific gene duplication. CKIδ was duplicated in the zebrafish *Danio rerio*, resulting in the so-called CKIδ-A and CKIδ-B. In the African clawed frog *Xenopus laevis*, a large genome duplication resulted in two CKIδ (L and S) and CKIε (L and S) genes. DBT sequences from protostomian representatives (the firebrat *Thermobia domestica*, the honey bee *Apis mellifera*, the marmorated sting bud *Halyomorpha halys*, the fruit fly *Drosophila melanogaster*, and the horseshoe crab *Limulus polyphemus*) were used as outgroups. The tree was inferred using RAxML maximum likelihood of 31 protein sequences using Geneious 11 software (Biomatters). Bootstrap support was calculated using 100 replications.

In our comprehensive analysis, we identified and further analyzed DBT sequences from 55 species representing 20 insect orders and 9 deuterostomian classes (Figure 3, Supplementary Figures S2–S7, and Supplementary Table S2). Whereas only one *dbt* gene was found in all analyzed insects, up to as many as three *dbt* paralogs were identified in the zebrafish *Danio* and four paralogs in the clawed frog *Xenopus*. In mammals, reptiles, and birds, two paralogous genes, CKIε and CKIδ, are known. Similarly, in the sea lamprey *Petromyzon marinus*, two *dbt*-like genes were discovered; however, a detailed sequence comparison indicates that these *dbt*-like genes result from lamprey-specific gene duplication (Figure 4). The CKIε/CKIδ separation is observed in sharks, rays, and fishes, and thus seems to be a result of gene duplication specific to Gnathostomata. The second duplication of CKIδ led to two CKIδ genes present in the zebrafish *Danio rerio* and probably a similar but independent (*Xenopus*-specific) duplication happened in the ancestor of the clawed frog *Xenopus laevis*. In addition to gene duplications and quadruplications, a various number of protein isoforms can be produced in some organisms from individual genes as a result of alternative splicing (see the C-terminal tail analysis below).

### NKRQK region and bride of DBT

The NKRQK motif (positions 220–224 in *Drosophila* DBT) within the catalytic domain distinguishing *D. melanogaster* DBT from the mouse homologs (Figure 1) was identified in all analyzed dipteran insects (Figure 3). Apart from the desert locust *Locusta migratoria* (the sequence was confirmed by Sanger sequencing and only one *dbt* gene was identified in the genome), all non-dipteran species contain the TKRQK motif. Therefore, we compared the presence of NKRQK to known changes in the circadian clock setup, such as the presence of mCRY, loss of dTIM (Kotwica-Rolinska et al., 2022a), and transformation of BMAL to CYC (Meireles-Filho et al., 2006). The transition of BMAL, a transcription factor with a transactivation domain, to CYC, a transcription factor which lacks the transactivation domain, was identified exclusively in Cyclorrhapha, a subset of Diptera (Supplementary Figure S8). However, this change perfectly corresponds to the loss of mCRY and does not agree with the presence of NKRQK (Figure 3).

**FIGURE 5:**
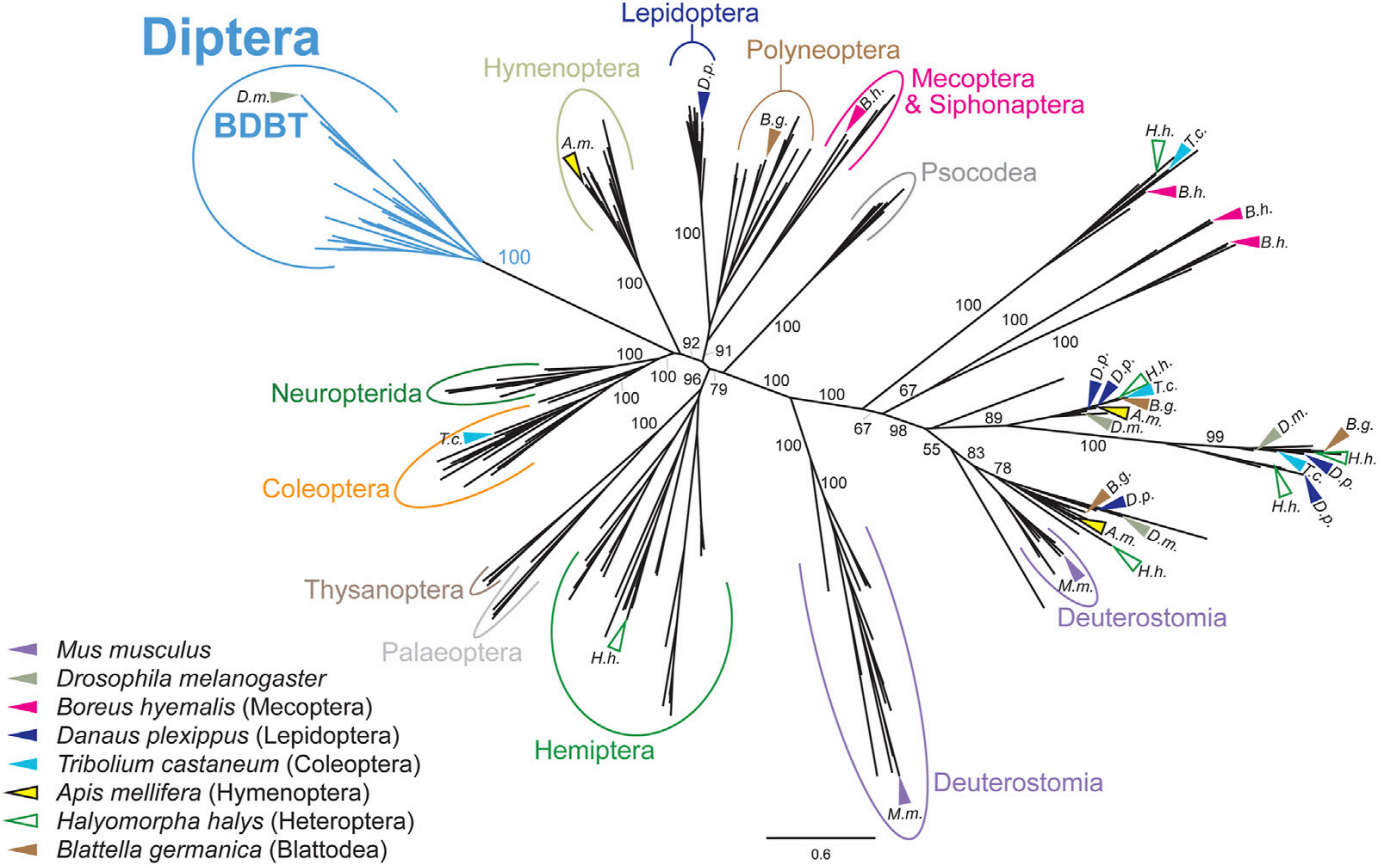
Phylogeny of FK506-binding proteins revealed a clear separation of dipteran BRIDE of DBT (BDBT) from all remaining clusters. Major insect orders are highlighted. Arrows indicate the position of sequences from representative species. FK506-binding proteins from Mecoptera and Siphonaptera do branch far away from Diptera. The tree was inferred using RAxML maximum likelihood of 280 protein sequences (final GAMMA-based score of the best tree −104683.7791) using Geneious 11 software (Biomatters). Bootstrap support was calculated using 100 replications.

Since none of the known changes in the clock setup correlated with the presence of the NKRQK motif in DBT, we analyzed the evolution of BDBT, a non-canonical FK506-binding protein interacting with DBT in *Drosophila* (Fan et al., 2013). First, we performed a phylogenetic analysis of available known FK506-binding proteins. The unrooted phylogenetic tree in Figure 5 (and the full tree version in Supplementary Figure S9) represents how various FK506-binding proteins evolved over time. Notably, dipteran BDBTs form a clear cluster that is separated from all remaining proteins. Furthermore, FK-506 binding proteins from Mecoptera (Scorpionflies) and Siphonaptera (Fleas), the closest relatives of Diptera, do not cluster with BDBT. Therefore, BDBT has been so heavily modified in Diptera that we cannot reliably identify the corresponding BDBT in any non-dipteran insect. Interestingly, in Diptera, the rise of the BDBT gene correlates with the transition from TKRQK to NKRQK motifs. The only other occurrence of the NKRQK motif was observed in *Locusta* (Orthoptera), whereas the sister polyneopteran lineages (termites, roaches, phasmids, Mantophasmatodea) contain TKRQK. However, FK-506 binding proteins of all polyneopteran lineages (including Orthoptera) branch together independently of TKRQK to NKRQK motifs.

### The C-terminal tail of CKI

The well-established impact of the C-terminal tail on the priming capacity of CKIδ splice isoforms in mice (Narasimamurthy and Virshup, 2021) prompted us to explore the C-terminal tail variability in the identified DBT/CKI dataset. The *in silico* predicted phosphorylation pattern was depicted for probabilities >0.5 (Figure 3 and Supplementary Figures S2–S7). When compared to the kinase domain, the C-terminal tails are the most variable parts of the proteins. Somewhat similar phosphorylation patterns and comparable lengths are found among C-terminal tails in vertebrates. In insects, however, the length and sequence of the C-terminal tails are remarkably variable. The shortest tails were identified in Lepidoptera (in several species only around 50 amino acids), whereas the longest tail in the *Anopheles* mosquito exceeded 250 amino acids (Figure 3). Putative phosphorylation was more prevalent in the C part of the C-terminal tail; however, the predicted phosphorylation patterns were quite variable in insects.

### Alternative splicing of the C-terminal tail

**FIGURE 6:**
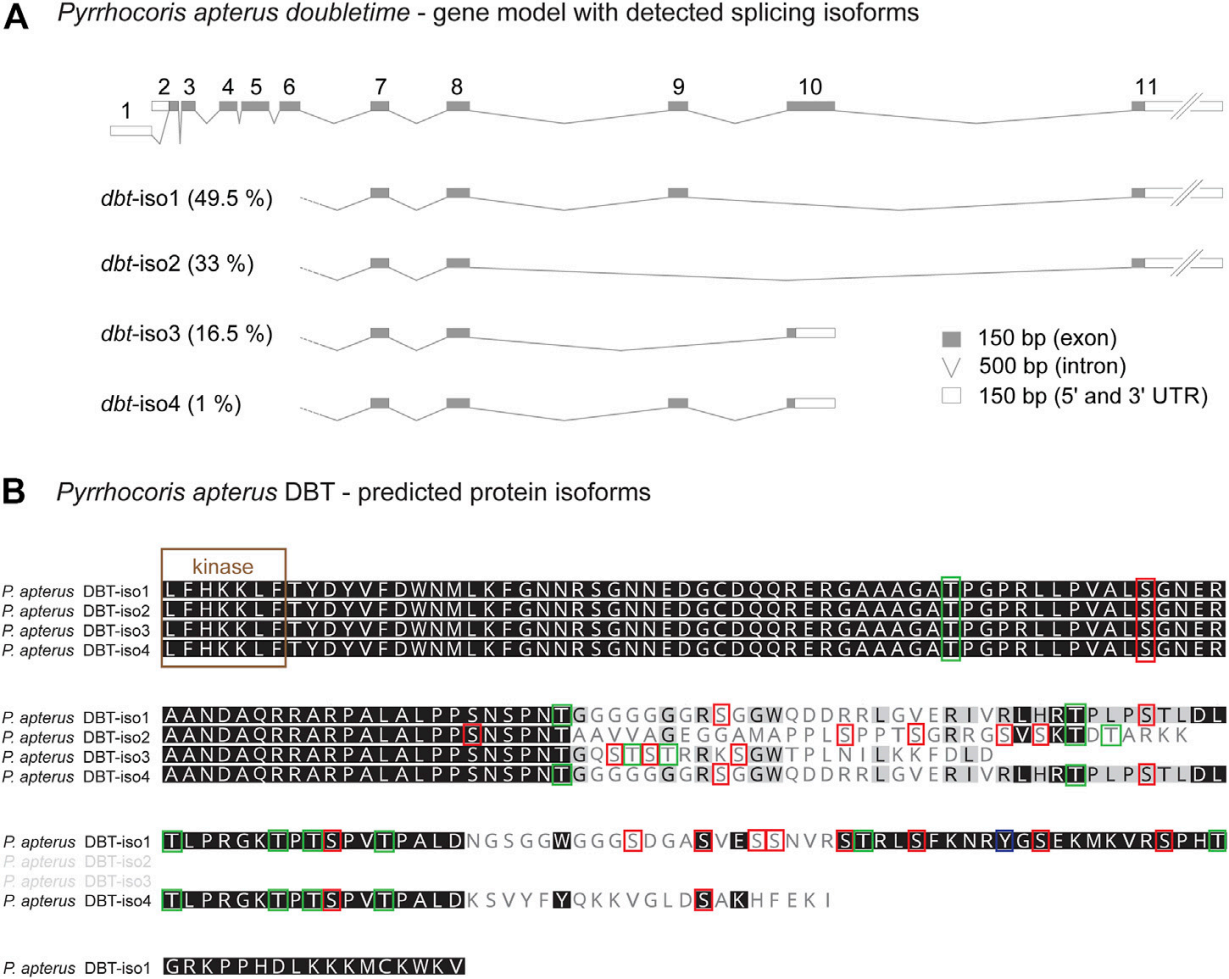
Alternative splicing of *doubletime* (*dbt*) gene in the linden bug *Pyrrhocoris apterus*. **(A)** A gene model illustrating the length of all *dbt* exons and introns. Two alternative transcription starts and splicing at the 5’end (exon 1 or 2, respectively) do not influence the predicted protein coding sequence. Alternative skipping of exons 9 and/or 10 results in four isoforms (*dbt*-iso1, 2, 3, 4). The expression level of each *dbt* isoform mRNA in the brain was detected from the Oxford nanopore transcriptome and depicted as % contribution to all *dbt* molecules (100%—all isoforms). **(B)** Protein alignment of the C-terminal tail illustrating the isoform-specific and variable regions (the more conserved residue the darker the color). Color rectangles refer to the theoretical prediction of phosphorylation pattern (>0.5; NetPhos-3.1) for serine (S), threonine (T), and tyrosine (Y).

Alternative splicing of mouse *CKIδ* transcripts affects the biochemical properties of resulting CKIδ protein isoforms. In all analyzed vertebrate species, CKIδ was alternatively spliced with impact on the predicted phosphorylation pattern in the terminal part of the C-terminal tail. In contrast, CKIε was alternatively spliced only in a few vertebrate species (Supplementary Figure S7). Alternative splicing was detected in *dbt* of many insects, including the mosquito *Anopheles albimanus* (Diptera), all analyzed beetles (Coleoptera), all analyzed butterflies/moths (Lepidoptera), all analyzed hymenopteran species (the honey bee *Apis mellifera*, the jewel wasp *Nasonia vitripennis*, and the turnip sawfly *Athalia rosae*), the pea aphid (*Acyrthosiphon pisum*), both analyzed species of Thysanoptera (the western flower thrips Frankliniella occidentalis and the melon thrips *Thrips palmi*), the silverleaf whitefly *Bemisia tabaci*, true bugs (Heteroptera: the water strider *Rhagovelia antilleana*, the kissing bug *Rhodnius prolixus*, the common bed bug *Cimex lectularius*, the brown marmorated stink bug *Halyomorpha halys*, and the linden bug *Pyrrhocoris apterus*), and the drywood termite *Cryptotermes secundus* (Supplementary Figures S3–S5). As a representative of the true bugs, having access to the linden bug *P. apterus* brain transcriptome obtained by Oxford Nanopore Technology, we analyzed the presence and abundance of all four identified *dbt* isoforms (Figure 6). The three most abundant isoforms comprising 99% of *dbt* transcripts encode proteins with predicted altered phosphorylation patterns. Thus, alternative splicing of *dbt* might serve as a regulatory step influencing and modulating the properties of DBT in some insects. In flies including *Drosophila*, however, *dbt* is an intronless gene. A similar intronless gene organization might exist in other insect species. However, the identification of only one transcript variant should be interpreted with caution. The reliable decision whether *dbt* gene contains introns requires a good non-fragmented genome assembly, ideally with well-annotated gene models. On the other hand, the identification of only one *dbt* isoform in the transcriptome of a particular species may reflect only shallow sequencing or might be affected by transcript assembly and post-sequencing processing.

### The C-terminal tail of DBT in diptera

**FIGURE 7:**
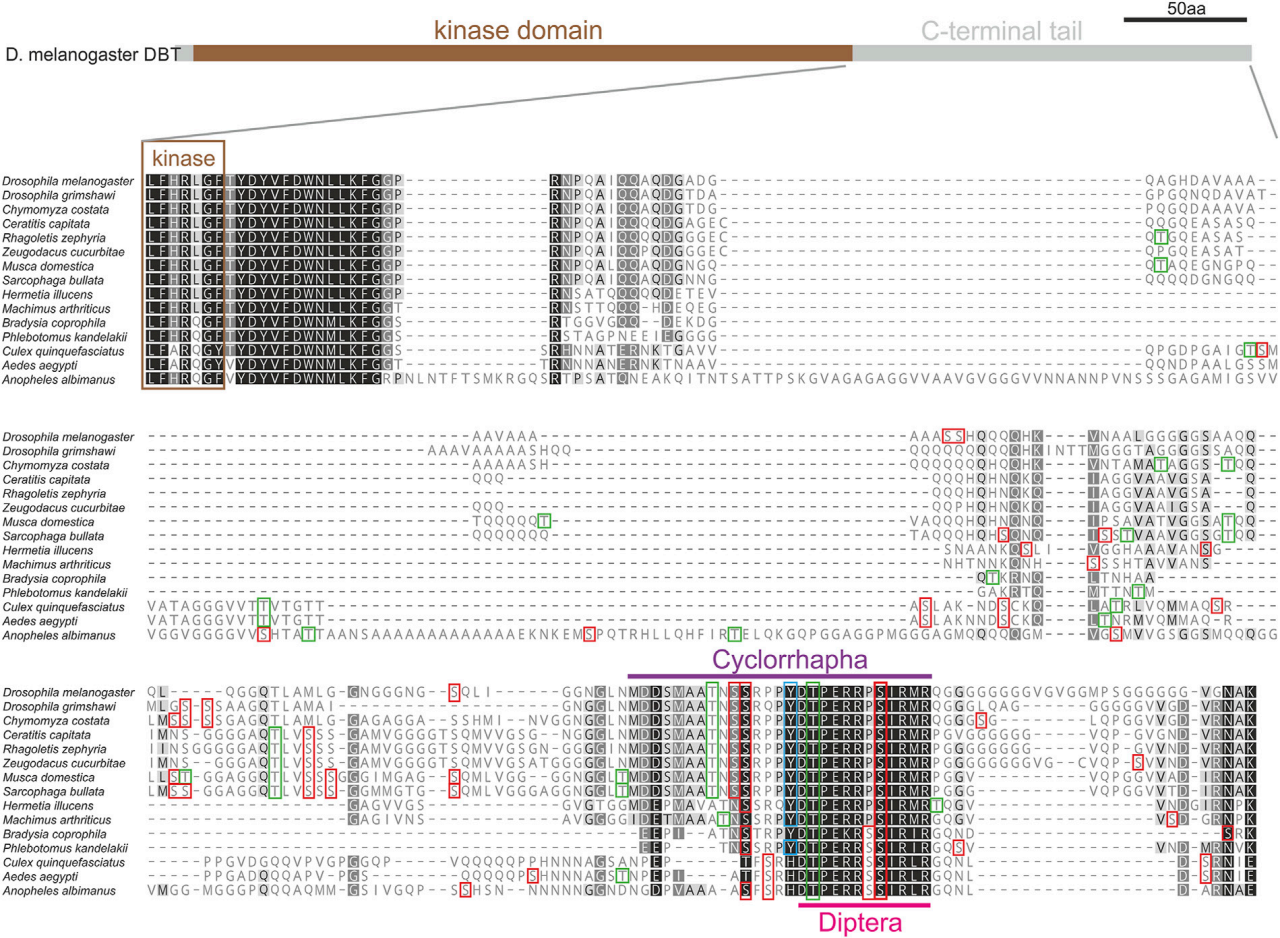
C-terminal tail of DBT in 15 representative species of Diptera. Protein alignment points to variable and conserved regions in the protein sequence (the more conserved the darker the color; a hyphen corresponds to a gap in the alignment). Color rectangles refer to the theoretical prediction of phosphorylation pattern (>0.5, NetPhos-3.1) for serin (S), threonine (T), and tyrosine (Y). The C-terminal tail contains a motif conserved in Diptera (light purple), which can be further extended in Cyclorhapha (Deep Purple).

The absence of introns in *dbt* genes of flies and the remarkable diversity of the C-terminal tail among flies and mosquitoes motivated a detailed comparison of this part of DBT in Diptera. Protein alignment of 15 species representing major dipteran lineages revealed a conserved region in the C-terminal tail, where a short 12 amino acid motif is identified in all dipteran species, and an even longer motif is shared among Cyclorrhapha (Figure 7). This conserved motif contains residues with high scores predicting their phosphorylation.

### Functional experiments in *Drosophila*

To test whether the identified sequence motifs in DBT have an impact on the function of the circadian clock *in vivo*, we applied reverse genetic tools to the fruit fly *D. melanogaster* and focused on two regions: 1) residue K224, which was established as important for temperature compensation in mammalian CKIδ (Shinohara et al., 2017), and which is also part of NKRQK motif (Figure 3), and 2) the C-terminal tail (Figure 7).

**FIGURE 8:**
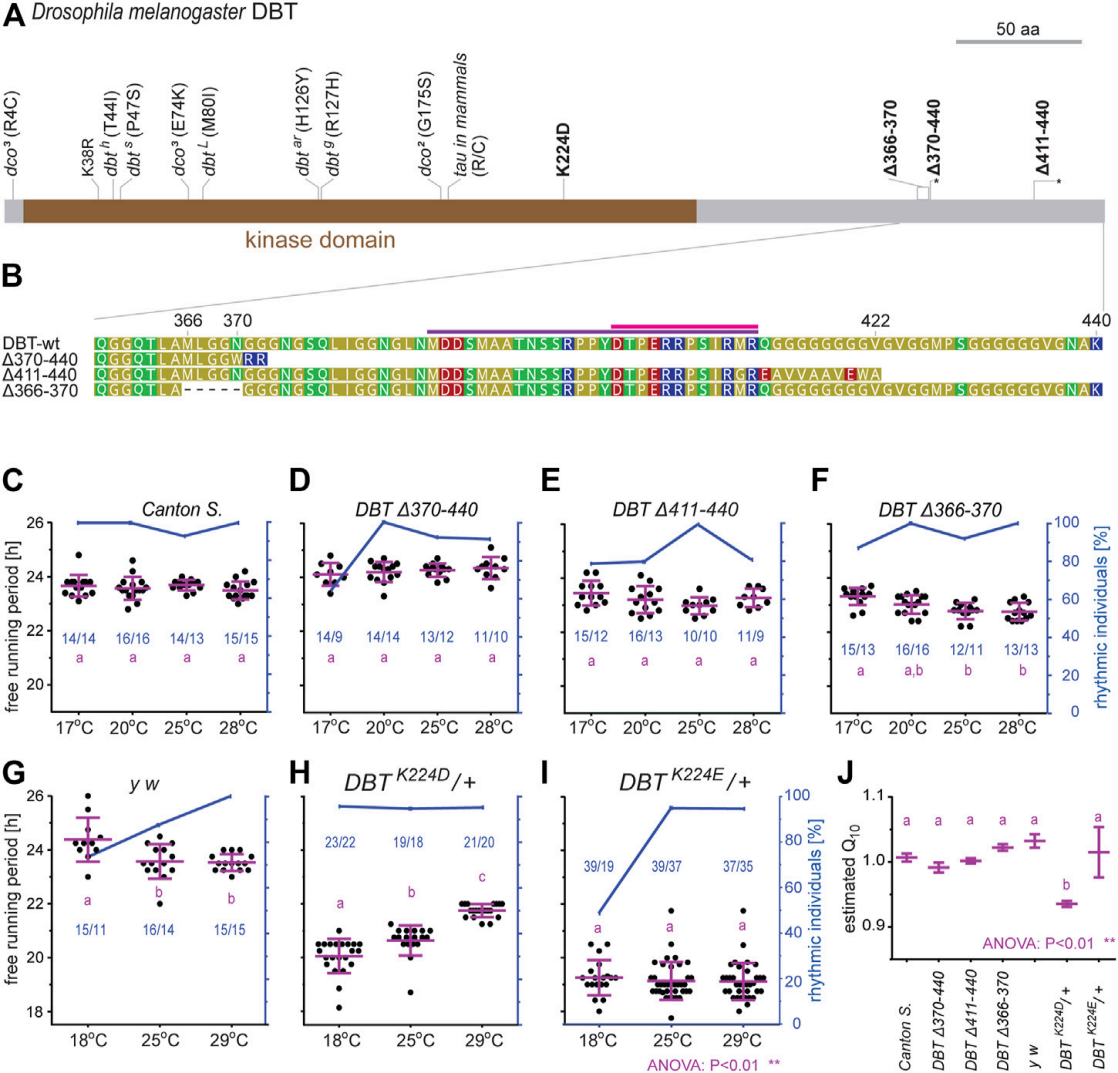
Functional analysis of new *dbt* mutants created in *D. melanogaster*. **(A)** A schematic depiction of DBT protein with highlighted mutations; here-created mutants are in bold, the asterisk in the scheme refers to a premature protein termination due to a frameshift. **(B)** Detail of the C-terminal tail in the wild type (wt) and three mutant lines. The dipteran and cyclorrhaphan motifs are highlighted by light and deep purple, respectively. **(C–I)** Circadian clock phenotype [each dot in panels **(C–I)** represents the free-running period tau, τ, of individual male flies] was recorded for 10 days in DD at the specified temperature. Blue dots connected with a blue line indicate the percentage of rhythmic individuals at a particular temperature. A scale indicating the percentage rhythmicity is on the right y-axis, the blue numbers in the chart represented n_total_/n_rhythmic_. **(J)** The temperature compensation depicted as Q_10_ was calculated from data presented in **(C–I)**. Magenta bars represent means ± SEM.

Three different C-terminal tail mutants were created, encompassing or bordering the conserved C-terminal tail domain (Figures 8A, B). All of them are homozygous viable and displayed only very mild, yet significant circadian phenotypes. The deletion of amino acids downstream of position 370, that is the part comprising conserved cyclorrhaphan and dipteran motifs, slightly extended τ when compared to controls (Figures 8C, D and Supplementary Table S4). Deletions of the very end of the C-terminal tail and frameshift (∆411–440) and a 15-bp in-frame deletion upstream of conserved motifs (∆366–370) mildly shortened τ (Figures 8E, F and Supplementary Table S4).

The basic lysine residue K224 was replaced by the acidic residues aspartic acid (D) or glutamic acid (E), respectively. In both cases, homozygous mutants were not viable, thus, heterozygous flies were analyzed. In both mutants, τ was significantly shorter compared to control flies. Moreover, these two mutants differ in their temperature compensation (Figures 8G–J). The K224D heterozygotes displayed a τ of ∼20 h at 18°C and lengthening of τ up to 22 h at higher temperatures (Q_10_ = 0.94) (Figures 8H, J). The K224E heterozygotes displayed an approximately 5-h faster clock compared to the wild type (τ ∼ 19) at all three tested temperatures and showed reduced rhythmicity at 18°C (Figures 8I, J). The shorter τ corresponded to earlier evening activity peaks in LD regimes in both mutant lines, K224D (Supplementary Figure S11) and K224E (Supplementary Figure S12), when compared to control *y w* flies (Supplementary Figure S10).

## Discussion

Recent remarkable progress in the genome and transcriptome sequencing allowed us to retrieve *dbt/CKI* genes from all major insect orders and from representative vertebrate lineages, and perform their sequence comparison. The available data point to several mutually independent CKI duplications observed in the deuterostomian lineage. Our analysis suggests that two *dbt*-homologous genes identified in *P. marinus* are the result of a *Petromyzon-*/lamprey-specific duplication, whereas duplication leading to the rise of *epsilon* and *delta* isoforms of CKI is dated to the common ancestor of Gnatohostomata. The complexity of the circadian clock setup in *Danio* and *Xenopus* has been shaped by independent genome duplications. In *Danio*, this gene duplication reflects complexity identified in many teleost species (Garcia-Concejo and Larhammar, 2021), however, in *Xenopus*, we see the outcome of a very recent species-limited genome duplication (Uno et al., 2013).

In addition to gene duplications, further diversity of CKIδ/ε is achieved *via* alternative splicing. Notably, CKIδ is alternatively spliced in all tetrapods (amphibia, reptiles, birds, and mammals), where the splicing influences the C-terminal tail of the protein including its putative phosphorylation pattern. In *Danio*, only one splice variant was identified for each CKIδ paralogue, thus, the protein diversity of CKIδ might be achieved by independent genes in this species.

The diversity of DBT sequences in insects is remarkable, and we suggest that the role of alternative splicing will most likely differ among various insect lineages. As we show in the linden bug *P. apterus*, not only are multiple splicing isoforms encoded by the *dbt* gene but three of them are expressed in the brain. Since *dbt* silencing results in a remarkable extension of τ in *P. apterus* (Kotwica-Rolinska et al., 2022a), functional tests of each splicing isoform would be an interesting research direction. Unfortunately, the isoform-specific exon 9 is only 159 nt long. Therefore, we are reaching technical limitations of ds RNA mediated interference, even though a 288 bp long dsRNA was successfully used to knock down isoform-specific transcripts (Bajgar et al., 2013). An alternative approach could utilize gene editing, a method established and used for circadian research in *P. apterus* (Kotwica-Rolinska et al., 2019; Kotwica-Rolinska et al., 2022b).

In some insects, however, no alternative splicing was detected and in certain lineages, such as flies, the *dbt* gene is intronless. Interestingly, our comprehensive analysis revealed three new *Drosophila* genus-specific CKI genes. To our knowledge, the role and function of these casein kinases are not established. Given the participation of both DBT and CKIα in the fruit fly circadian clock (Lam et al., 2018), one cannot rule out the involvement of here-identified CKI genes in the circadian clock. Although this is entirely speculative, the combination of multiple kinases encoded by independent genes would provide an alternative source to the isoform repertoire produced by alternative splicing from an individual gene in some other species.

Functional analysis of the C-terminal tail in *D. melanogaster* revealed negligible effects on rhythmicity or changes in τ. However, only three simple deletion mutants were created here, thus, a full evaluation of the role for the C-terminal tail in DBT is rather premature. The second set of mutants, K224 modifications, resulted in homozygous lethality, similar to known strong *dbt/dco* alleles disrupting the developmental function of CK1 during the pupal stage (Price et al., 1998; Zilian et al., 1999). The equivalent mammalian mutations are not homozygous lethal, presumably because CK1δ and CK1ε are able to complement each other. Heterozygous mutant K224D/+ and K224E/+ flies produced profound shortenings of τ, almost identical to homozygous CK1δ K224D mutant mice (Shinohara et al., 2017, Figures 8G–I). Acidic K224 substitutions presumably bypass the phospho relay embedded in the *per* ^*S*^ serine cluster (FASP serine cluster in mPER2), and immediately phosphorylate the respective serine in the PER phosphodegron. This is because in mammalian CK1, K224 (together with R178) forms an anion-binding site required for phosphorylation of primed substrates (Narasimamurthy and Virshup, 2021). In flies and mammals, phosphorylation of the *per* ^*S*^/FASP serine cluster delays PER degradation by preventing premature phosphorylation at the phosphodegron site, which would lead to rapid PER turnover (phosphoswitch). However, phosphorylation of multiple serine residues within the *per* ^*S*^/FASP cluster by DBT/CK1 requires a priming phosphorylation at a particular serine within each cluster. Binding of DBT/CK1 to this primed substrate requires the basic anion binding pocket formed by R178 and K224, which most likely can not form in acidic K224D and K224E mutants. Therefore, the short periods observed in K224D, K224E, and R178 (= hamster *tau* mutant: Ralph and Menaker, 1988; Lowrey et al., 2000) are presumably the consequence of impaired *per* ^*S*^/FASP region phosphorylation and the resulting acceleration of phosphorylation at the phosphodegron site. Moreover, the K224D mutation affects temperature compensation, both in flies and mouse organ cultures. However, the temperature-dependent lengthening of τ associated with the K224D mutation in flies is opposite to what was detected for *mPer2-luc* expression in suprachiasmatic nucleus (SCN) slices of homozygous K224D mice, which showed a shortening of τ with increasing temperature (Shinohara et al., 2017). The opposite temperature compensation phenotypes of K224D in flies and mice, as well as the lack of temperature-dependent period lengthening in the very similar K224E mutants, suggest that K224D overcompensation in flies is not simply caused by potential thermal instability of the K224D protein. Moreover, temperature overcompensation is not generally linked to decreased protein stability at higher temperatures (Giesecke et al., 2021). The phenotypic differences between the fly K224D and K224E mutants are surprising (temperature overcompensation in K224D and reduced rhythmicity at 18°C in K224E, Figure 8). These differences demonstrate that the two replacements have different consequences (apart from both shortening the free-running period), even though both introduce an acidic residue. Although very similar in structure, glutamic acid is slightly larger compared to aspartic acid, which may influence substrate binding in a temperature-dependent manner. In addition, the presence of an Asparagine (N) at position 220 in fly DBT, as opposed to a Threonine (T) at this position in CKIδ, might contribute to the opposite temperature compensation phenotypes of K224D mutants in flies and mice. Replacing the fly Asparagine (N220) with a Threonine would be interesting, not only in the light of temperature compensation differences, but also in the context of recently published autophosphorylation of Threonine, which is the preferred amino acid in corresponding positions in the majority of homologous kinases (Cullati et al., 2022). Secondly, the possible interaction with BDBT in the fruitfly might be an interesting and experimentally testable explanation for the altered temperature compensation in K224D. On the other hand, the NKRQK motif arose independently in *Locusta* and other orthopteran species and, given the diversity of FK-506 binding proteins, can hardly be interpreted as BDBT-dependent modification. Moreover, given the broad range of targets phosphorylated by DBT and even its non-catalytic role in the circadian clock (Yu et al., 2009), the mechanistic model might be quite complex.

## Data Availability

The original contributions presented in the study are included in the article/Supplementary Material, further inquiries can be directed to the corresponding authors.
