# Supplementary figures and images for: Evolution of casein kinase 1 and functional analysis of new doubletime mutants in Drosophila
Source: Front Physiol. 2022 Dec 14;13:1062632. doi: 10.3389/fphys.2022.1062632 (PMC9794997; doi:10.3389/fphys.2022.1062632)

### dbtK224E + 18

TC20CtM078C17-TC20CtM078C32 (n=16 days=2.9)

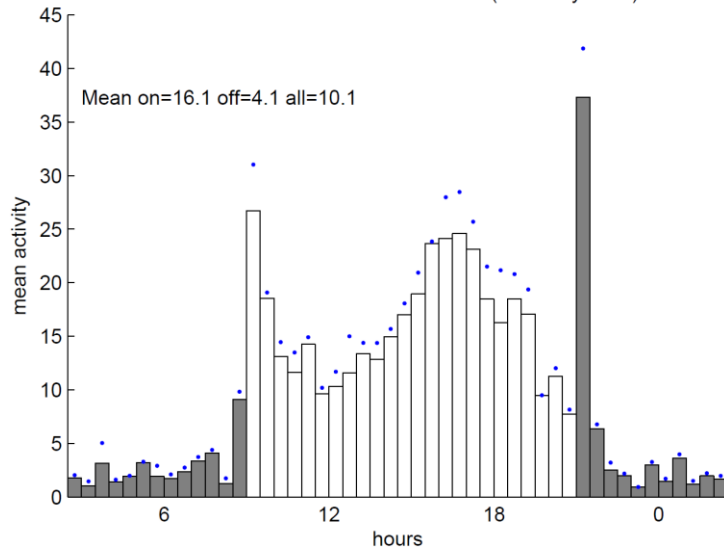

### dbtK224E + 25

TC20CtM017C17-TC20CtM017C32 (n=16 days=2.9)

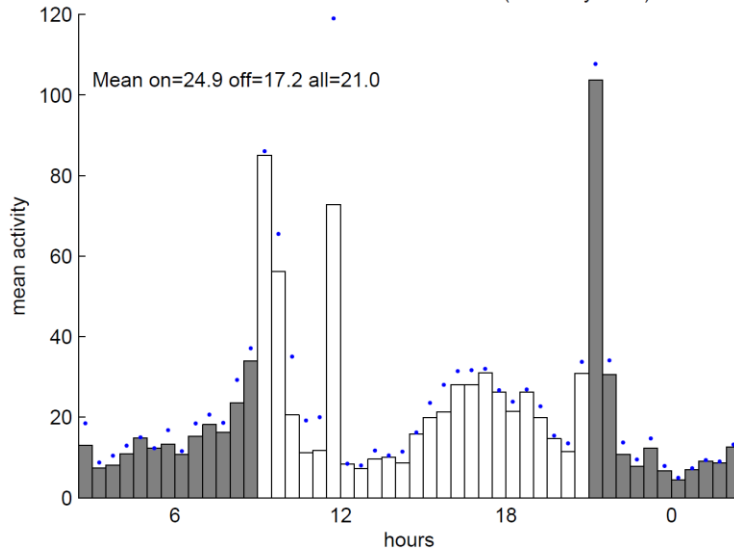

### dbtK224E + 29

TC20CtM018C17-TC20CtM018C32 (n=15 days=2.9)

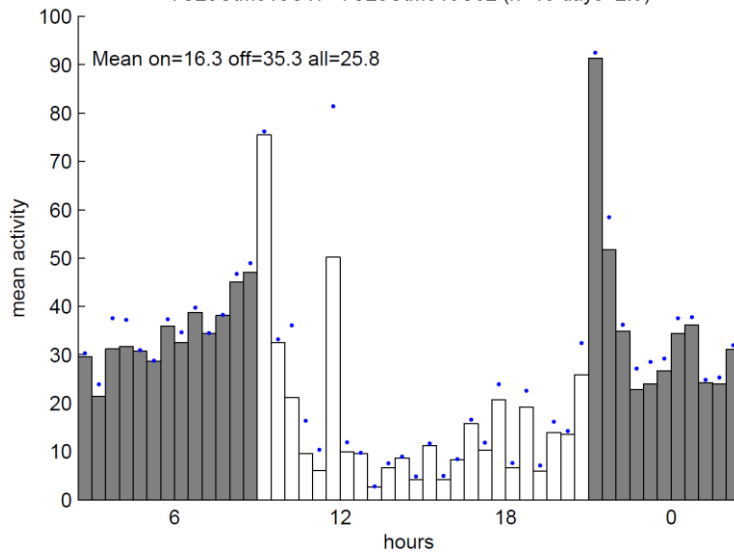

Supplement: Supplementary file 1 [file Image12.PDF]

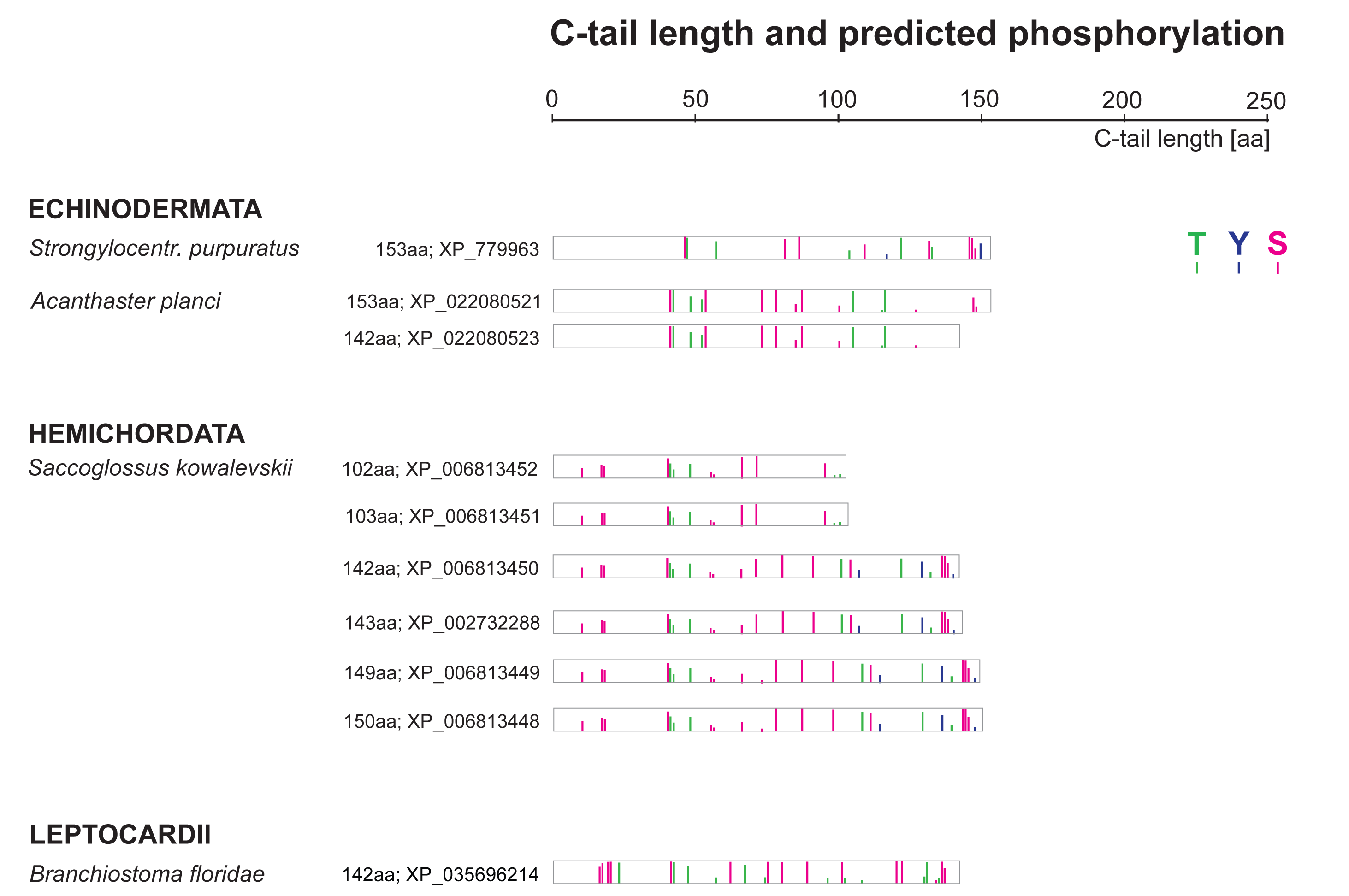

Supplement: Supplementary file 2 [file Image6.TIF]

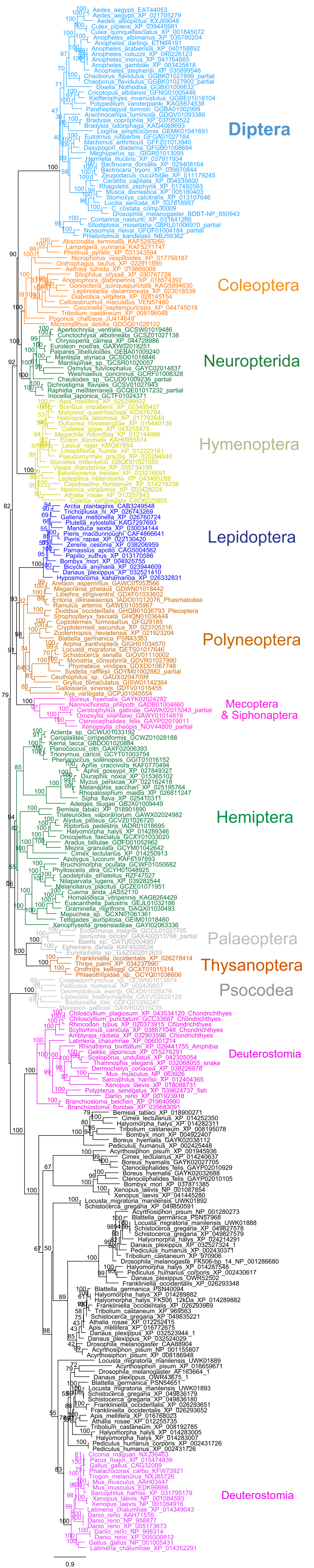

Supplement: Supplementary file 4 [file Image9.PDF]

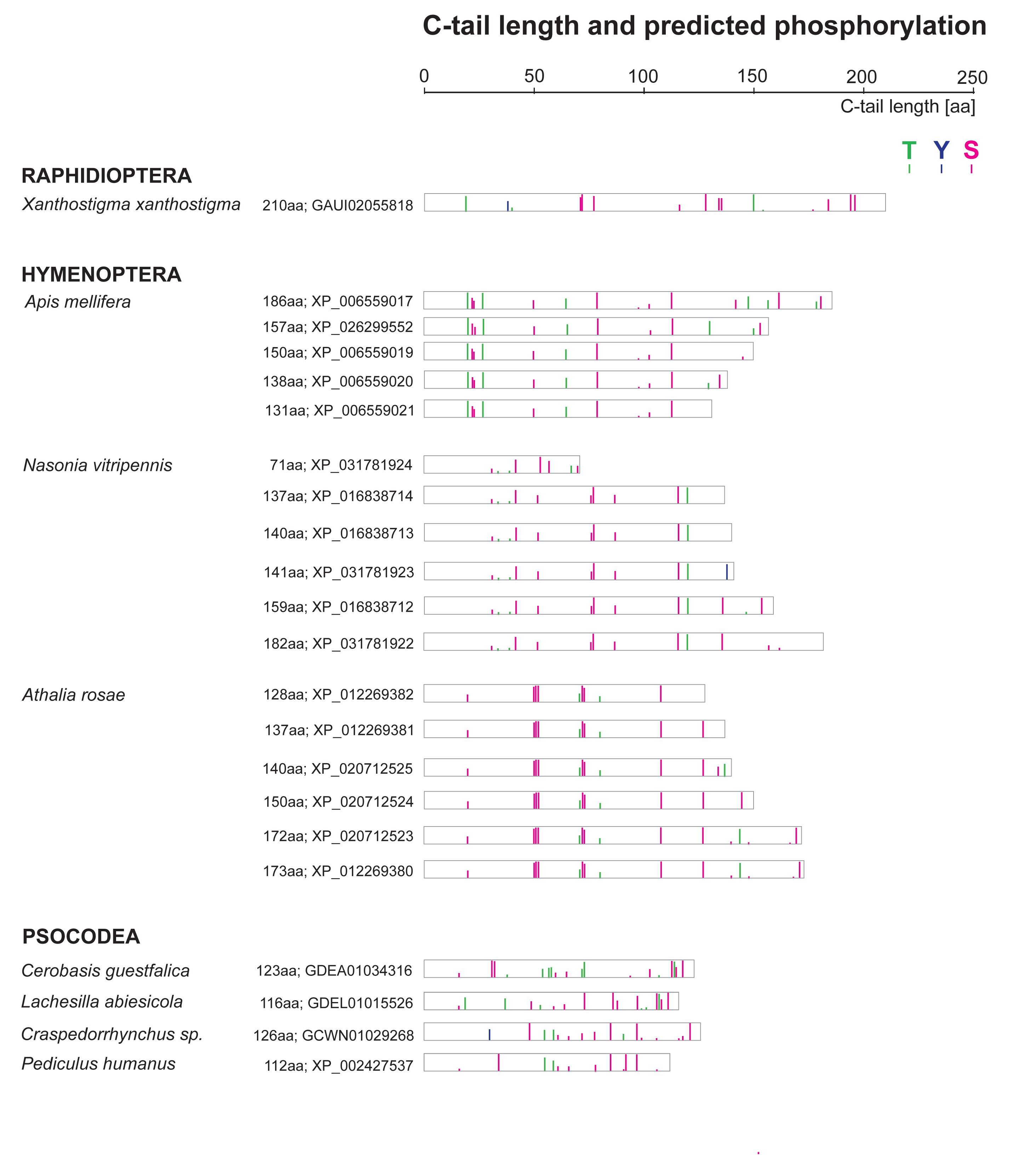

Supplement: Supplementary file 5 [file Image3.TIF]

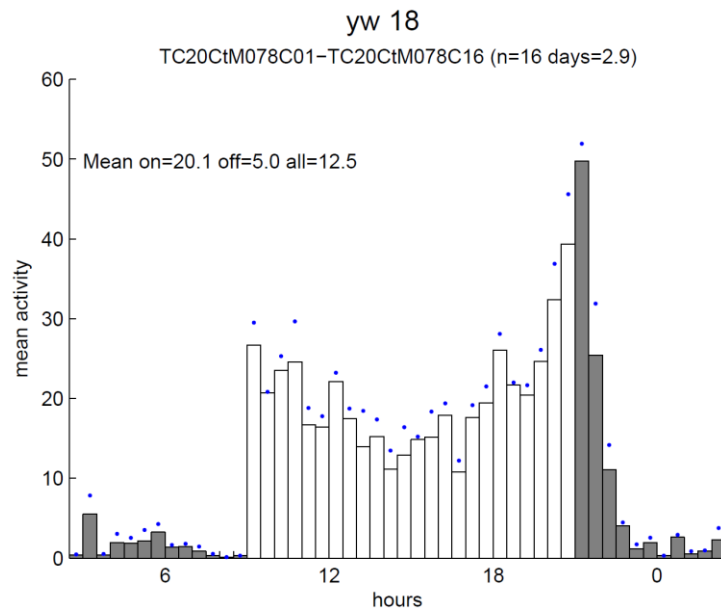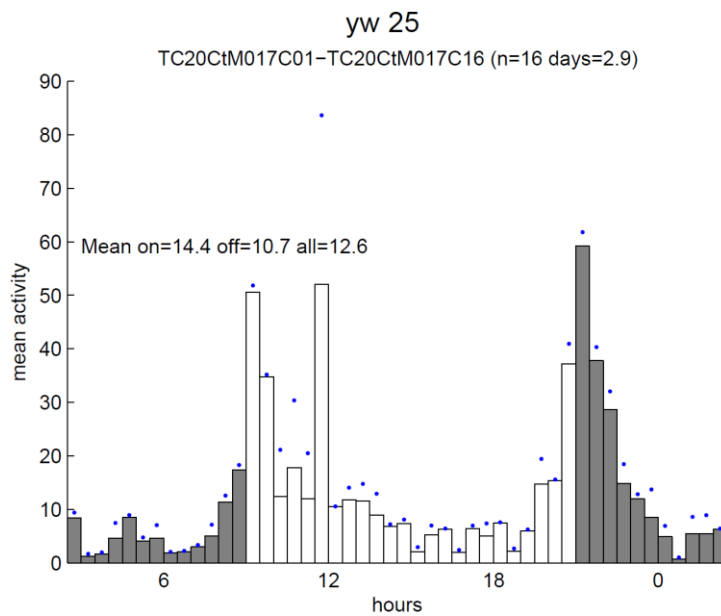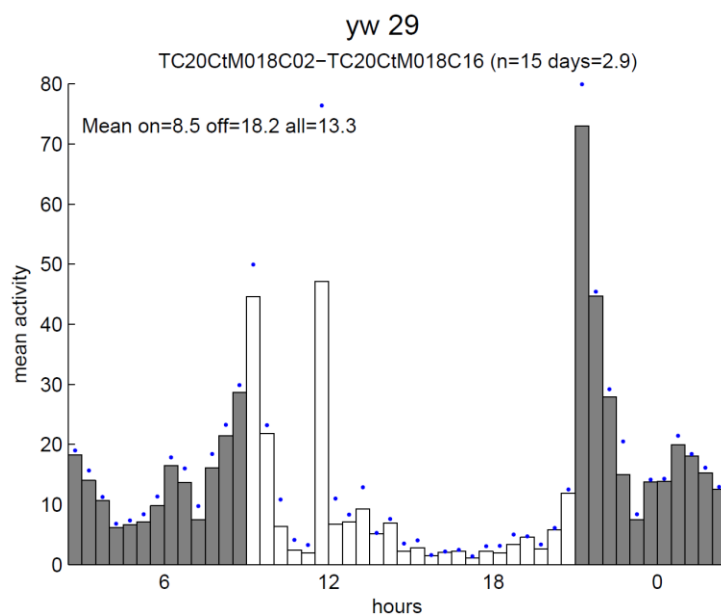

Supplement: Supplementary file 6 [file Image10.PDF]

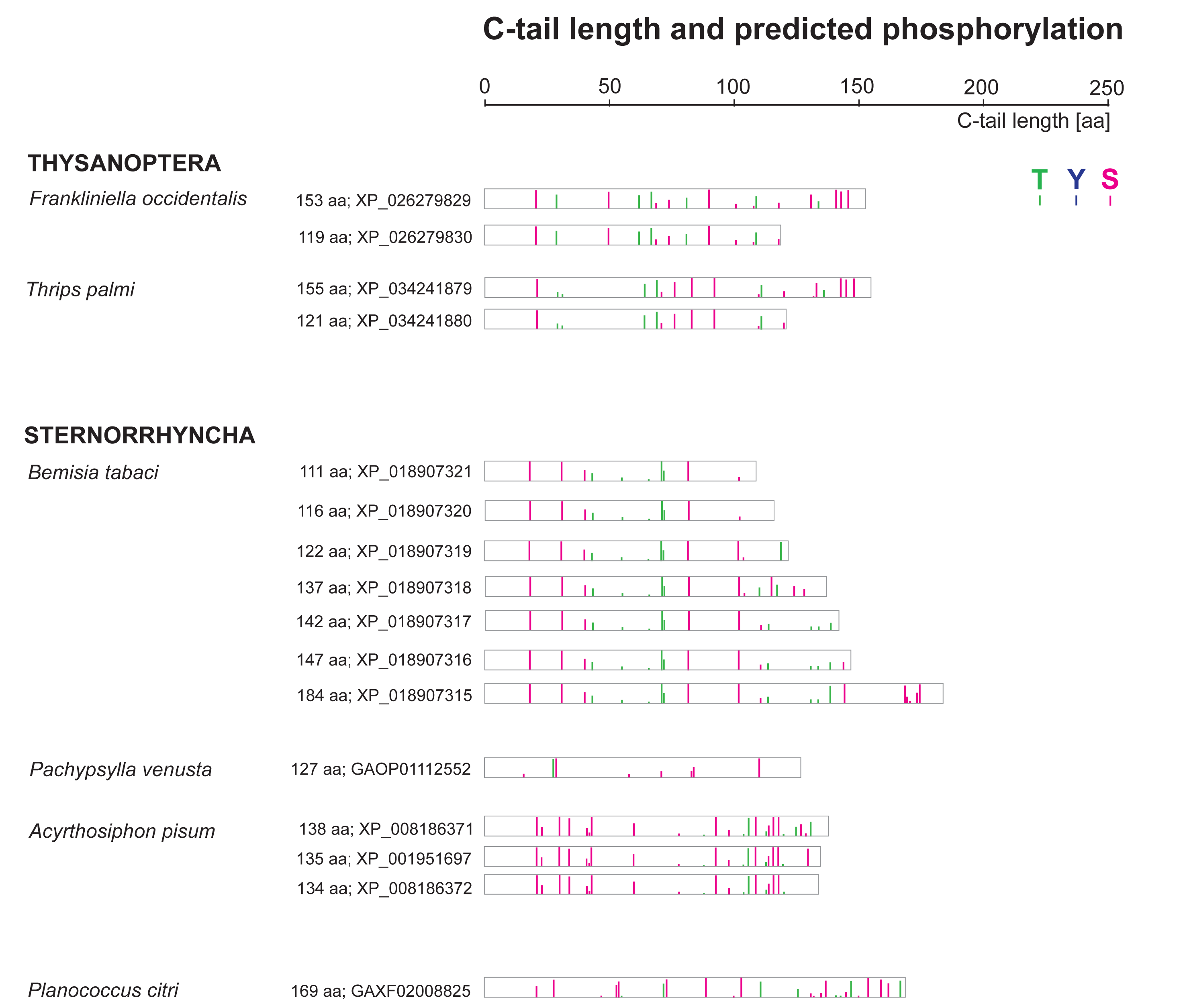

Supplement: Supplementary file 7 [file Image4.TIF]

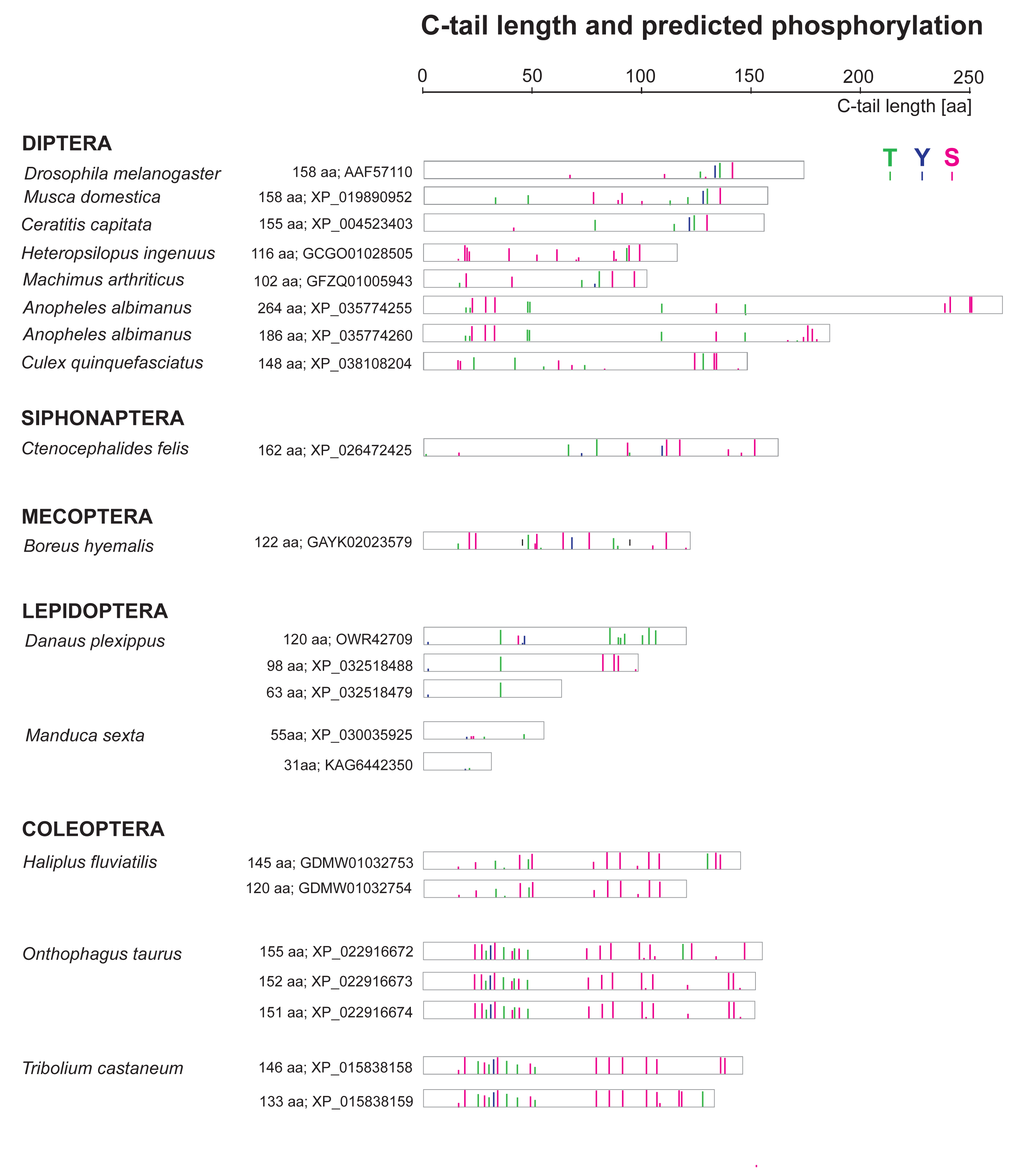

Supplement: Supplementary file 8 [file Image2.TIF]

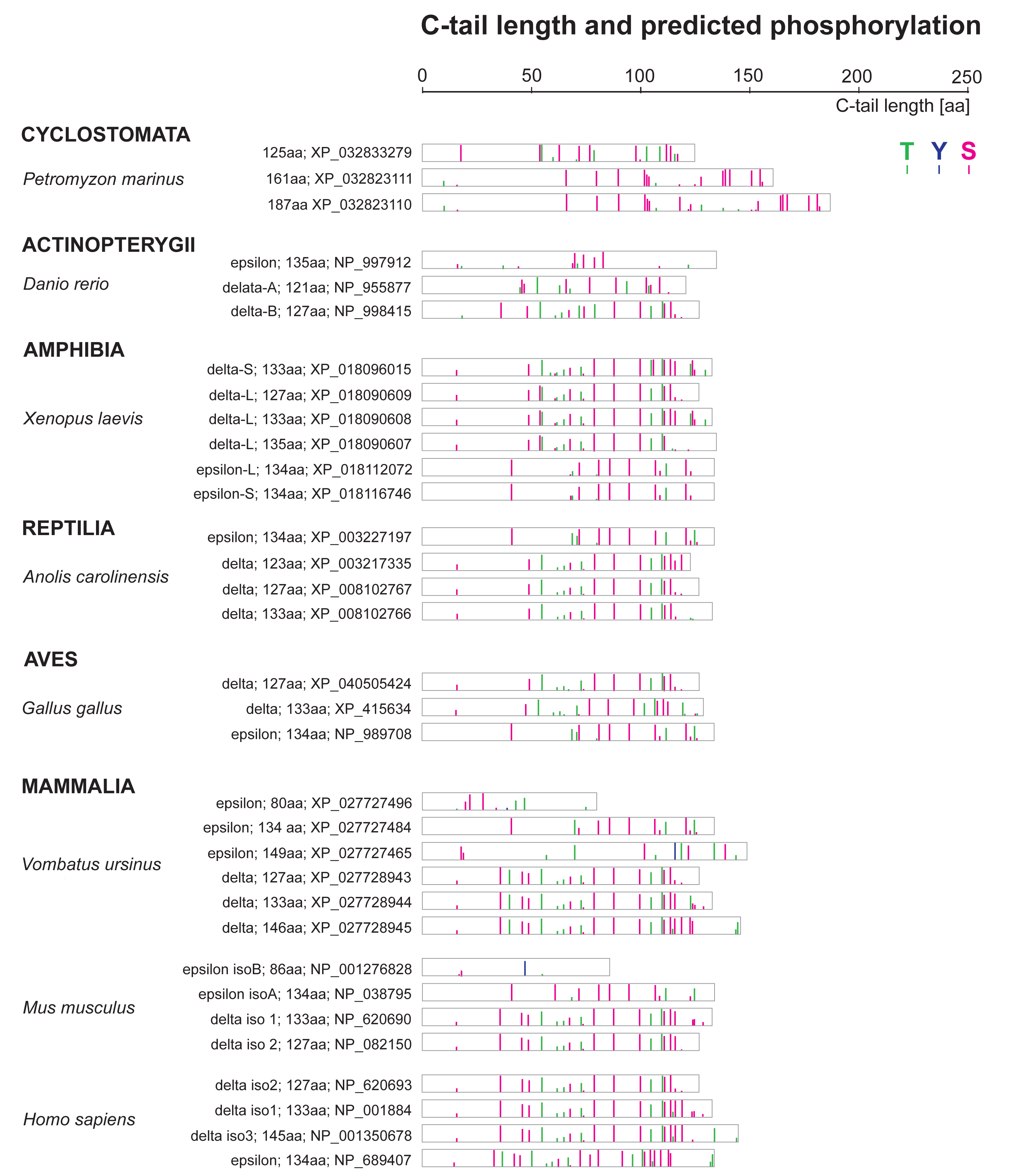

Supplement: Supplementary file 9 [file Image7.TIF]

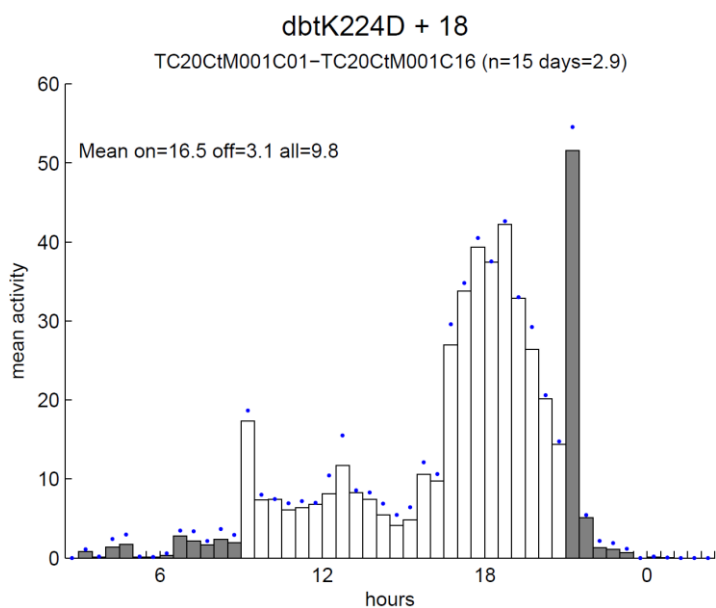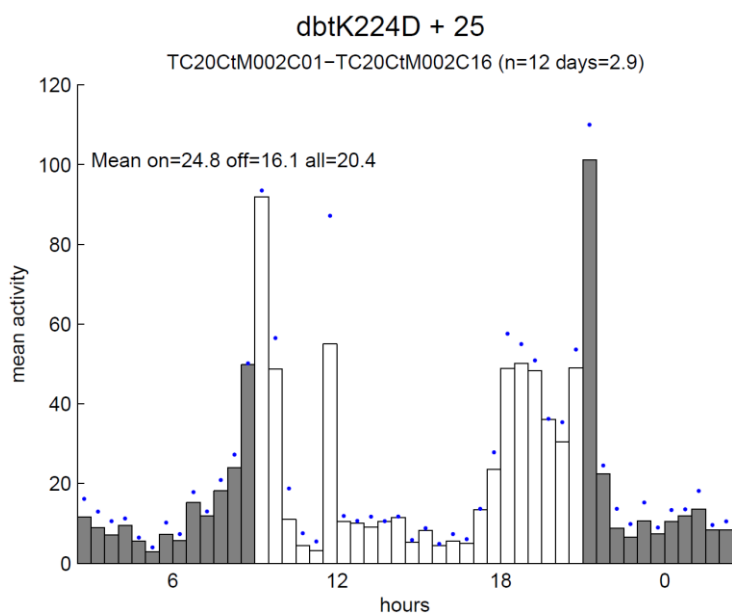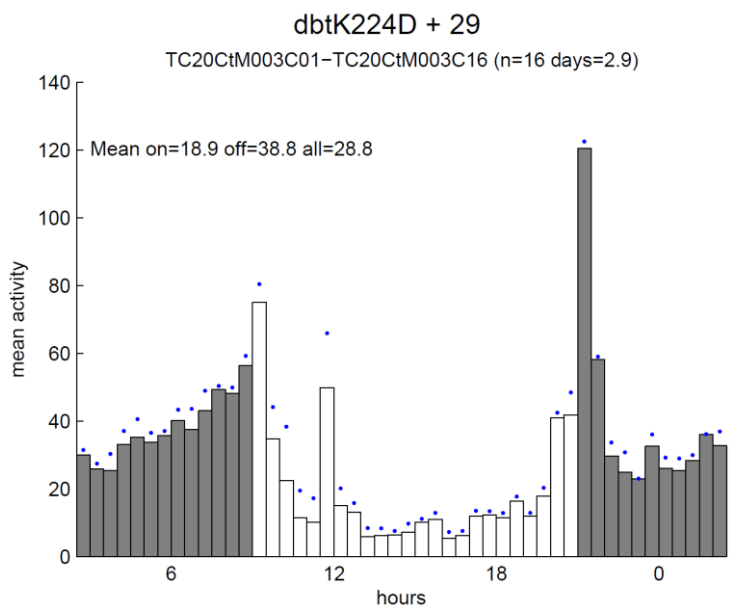

Supplement: Supplementary file 14 [file Image11.PDF]

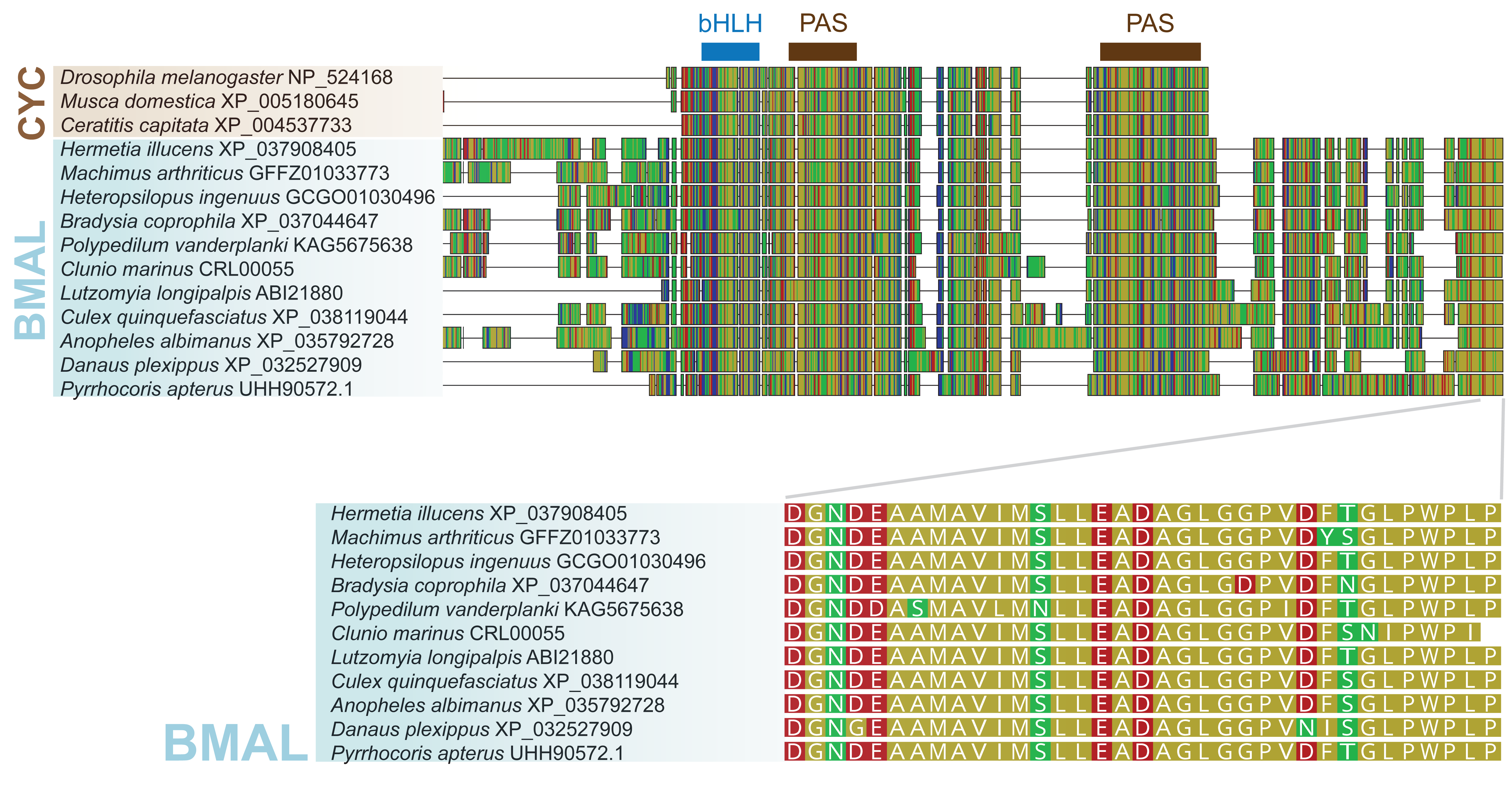

Supplement: Supplementary file 15 [file Image8.TIF]

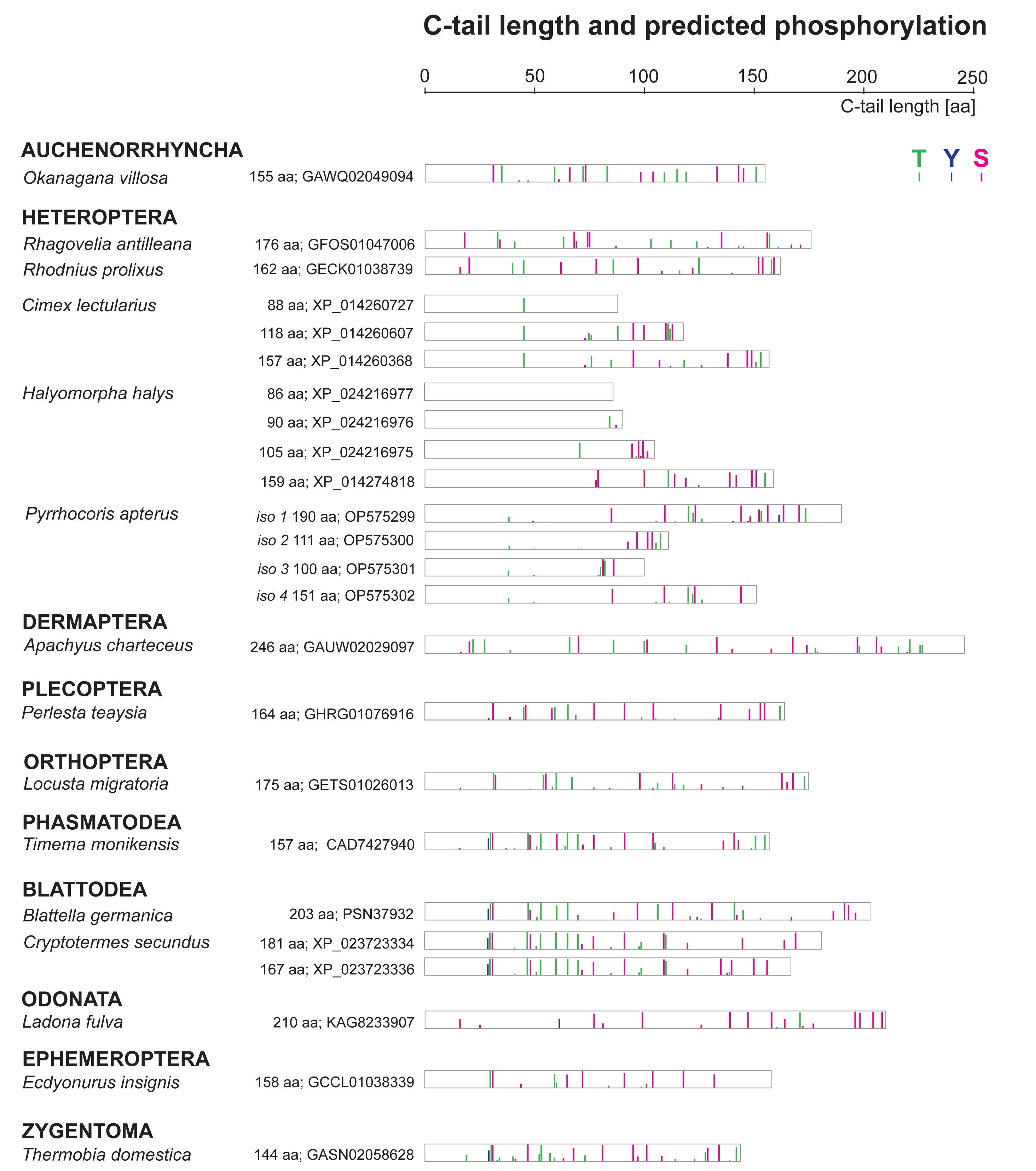

Supplement: Supplementary file 16 [file Image5.TIF]
