# Supplementary material for: Evolution of casein kinase 1 and functional analysis of new doubletime mutants in Drosophila
Source: Front Physiol. 2022 Dec 14;13:1062632. doi: 10.3389/fphys.2022.1062632 (PMC9794997; doi:10.3389/fphys.2022.1062632)
Supplement: Supplementary file 3 [file Table1.DOCX]

| **Species/group** | **Gene** | **Protein acc number**  **isoforms** | **note** |
| --- | --- | --- | --- |
| ***Drosophila melanogaster***  Insecta; Holometabola; Diptera; Brachycera; Cyclorrhapha; Drosophilidae | 1 | AAN09313 | CKIalpha |
|  | 1 | AAL68089 / AT17410p | CKIalpha-like I, *Drosophila* genus unique |
|  | 1 | NP_649536/CG12147 | CKIalpha-like II, *Drosophila* genus unique |
|  | 1 | NP_608697/CG9962 | *Drosophila* genus unique CKI |
|  | 1 | NP_732123/gilgamesh_isoA | gilgammesh/CKIgamma  (splicing isoforms) |
|  |  | AAF55294/gilgamesh_isoB |  |
|  |  | NP_524941/gilgamesh_isoC |  |
|  |  | AAN13704/gilgamesh_isoD |  |
|  |  | NP_788683/gilgamesh_isoE |  |
|  |  | NP_001014628/gilgamesh_isoF |  |
|  |  | NP_001014627/gilgamesh_isoG |  |
|  |  | NP_001163628/gilgamesh_isoI |  |
|  |  | NP_001247137/gilgamesh_isoJ |  |
|  |  | NP_001247138/gilgamesh_isoK |  |
|  |  | NP_001262624/gilgamesh_isoL |  |
|  |  | NP_001262625/gilgamesh_isoM |  |
|  |  | NP_001369018/gilgamesh_isoN |  |
|  |  | NP_001369019/gilgamesh_isoO |  |
|  |  | NP_001369020/gilgamesh_isoP |  |
|  | 1 | NP_572794/CG2577 | *Drosophila* genus unique CKI |
|  | 1 | NP_726577 | asator / tau-tubulin kinase 1  (splicing isoforms) |
|  |  | AAY55135 |  |
| ***Drosophila suzulii***  Insecta; Holometabola; Diptera; Brachycera; Cyclorrhapha; Drosophilidae | 1 | XP_016923997 | CKIalpha |
|  | 1 | XP_016928528 | CKIalpha-like I, *Drosophila* genus unique |
|  | 1 | XP_016931834 | CKIalpha-like II, *Drosophila* genus unique |
|  | 1 | XP_016932540 | *Drosophila* genus unique CKI |
|  | 1 | XP_036673801 | gilgammesh/CKIgamma |
|  | 1 | XP_016923903 | *Drosophila* genus unique CKI |
| ***Drosophila willistoni***  Insecta; Holometabola; Diptera; Brachycera; Cyclorrhapha; Drosophilidae | 1 | XP_002070931 | CKIalpha |
|  | 1 | GJOF01005569 | CKIalpha-like I, *Drosophila* genus unique |
|  | 1 | EDW83428 1 | CKIalpha-like II, *Drosophila* genus unique |
|  | 1 | XP_002066575 | *Drosophila* genus unique CKI |
|  | 1 | XP_023035705 | gilgammesh/CKIgamma |
|  | 1 | GJOF01002639 | *Drosophila* genus unique CKI |
| ***Drosophila virilis***  Insecta; Holometabola; Diptera; Brachycera; Cyclorrhapha; Drosophilidae | **1** | **XP_002058127** | CKIalpha |
|  | 1 | XP_002058128 | CKIalpha |
|  | 1 | XP_002048901 | CKIalpha-like I, *Drosophila* genus unique |
|  | 1 | KRF79126 | CKIalpha-like II, *Drosophila* genus unique |
|  | 1 | XP_002052097 | *Drosophila* genus unique CKI |
|  | 1 | XP_032289459 | gilgammesh/CKIgamma |
|  | 1 | XP_002057057 | *Drosophila* genus unique CKI |
| ***Drosophila grimshawi***  Insecta; Holometabola; Diptera; Brachycera; Cyclorrhapha; Drosophilidae | 1 | XP_001991332 | CKIalpha |
|  | 1 | XP_001991333 | CKIalpha |
|  | 1 | XP_001989259 | CKIalpha-like I, *Drosophila* genus unique |
|  | 1 | XP_001994022 | CKIalpha-like II, *Drosophila* genus unique |
|  | 1 | XP_001990762 | gilgammesh/CKIgamma |
|  | 1 | XP_001995815 | *Drosophila* genus unique CKI |
| ***Ceratitis capitata***  Insecta; Holometabola; Diptera; Brachycera; Cyclorrhapha, Tephritidae | 1 | XP_004536733 | CKIalpha |
|  | 1 | XP_020718038 | gilgammesh/CKIgamma  splicing isoforms |
|  |  | XP_020718035 |  |
|  |  | XP_023158495 |  |
|  |  | XP_023158494 |  |
|  |  | XP_020718036 |  |
|  |  | XP_023158492 |  |
|  |  | XP_023158491 |  |
|  |  | XP_023158493 |  |
|  | 1 | XP_012160860 | asator / tau-tubulin kinase |
| ***Musca domestica***  Insecta; Holometabola; Diptera; Brachycera; Cyclorrhapha, Muscidae | 1 | XP_005175423 | CKIalpha |
|  | 1 | XP_019894430 | gilgammesh/CKIgamma  splicing isoforms |
|  |  | XP_019894431 |  |
|  |  | XP_019894432 |  |
|  |  | XP_019894433 |  |
|  |  | XP_01989343 | asator / tau-tubulin kinase |
| ***Anopheles albimatus***  Insecta; Holometabola; Diptera; Nematocera; Culicidae | 1 | XP_035784016 | CKIalpha |
|  | 1 | XP_035782093 | gilgammesh/CKIgamma  splicing isoforms |
|  |  | XP_035782080 |  |
|  |  | XP_035782088 |  |
|  |  | XP_035782089 |  |
|  |  | XP_035782092 |  |
|  |  | XP_035782086 |  |
|  |  | XP_035782091 |  |
|  | 1 | XP_035784891 | asator / tau-tubulin kinase |
| ***Culex quinquefasciatus***  Insecta; Holometabola; Diptera; Nematocera; Culicidae | 1 | EDS27991 | CKIalpha  splicing isoforms |
|  |  | XP_038111213 |  |
|  | 1 | XP_038116328 | gilgammesh/CKIgamma  splicing isoforms |
|  |  | XP_038116330 |  |
|  |  | XP_038116331 |  |
|  | 1 | XP_038116441 | asator / tau-tubulin kinase |
| ***Danaus plexippus***  Insecta; Holometabola;  Lepidoptera; Ditrysia;  Nymphalidae | 1 | XP_032523226 | CKIalpha |
|  | 1 | XP_032525620 | asator / tau-tubulin kinase  (splicing isoforms) |
|  | 1 | OWR52845 |  |
| ***Bombyx mori*** Insecta; Holometabola;  Lepidoptera; | 1 | NP_001037287 | CKIalpha |
| ***Halyomorpha halys***  Insecta; Paraneoptera; Hemiptera; Heteroptera; Pentatomomorpha; | 1 | XP_014272268 | CKIalpha |
|  | 1 | XP_024217897 | gilgammesh/CKIgamma  splicing isoforms |
|  |  | XP_024217016 |  |
|  | 1 | XP_014270770 | asator / tau-tubulin kinase  (splicing isoforms) |
|  |  | XP_014270778 |  |
| ***Thermobia domestica***  Insecta; Zygentoma; Lepismatidae; | 1 | GASN02046539 | CKIalpha |
|  | 1 | GASN02049146 | gilgammesh/CKIgamma |
|  | 1 | GASN02059192 | asator / tau-tubulin kinase |
| ***Strongylocentrotus purpuratus***  Echinodermata; Echinoidea; Camarodonta; Strongylocentrotidae | 1 | XP_786391 | CKIalpha  splicing isoforms |
|  |  | XP_030841481 |  |
|  | 1 | XP_783545 | CKIalpha-like |
|  | 1 | XP_779900 | CKIgamma |
| ***Branchiostoma* *floridae***  Chordata; Leptocardii; Branchiostomidae | 1 | XP_035672527 | CKIalpha |
|  | 1 | XP_035666971 | CKIgamma |
| ***Petromyzon marinus***  Metazoa; Chordata; Vertebrata; Cyclostomata; Hyperoartia; | 1 | XP_032806746 | CKIalpha  splicing isoforms |
|  |  | XP_032806745 |  |
|  | 2 | XP_032821058 | asator / tau-tubulin kinase |
|  |  | XP_032819888 | asator / tau-tubulin kinase |
| ***Mus musculus***  Chordata; Vertebrata; Tetrapoda; Mammalia; Rodentia; | 1 | EDL09767 | CKIalpha  splicing isoforms |
|  |  | NP_001344429 |  |
|  |  | NP_001344427 |  |
|  |  | NP_001344428 |  |
|  | 3 | NP_775277 | CKIgamma-1 |
|  |  | NP_001153063 | CKIgamma-2 |
|  |  | EDL09899 | CKIgamma-3  splicing isoforms |
|  |  | EDL09900 |  |
|  |  | EDL28020 | tau tubulin kinase 2 |
|  |  | BAB62004 | Tau-tubulin kinase |
|  |  | XP_006523508 | tau-tubulin kinase 1 isoform X3 |
