# Supplementary material for: Evolution of casein kinase 1 and functional analysis of new doubletime mutants in Drosophila
Source: Front Physiol. 2022 Dec 14;13:1062632. doi: 10.3389/fphys.2022.1062632 (PMC9794997; doi:10.3389/fphys.2022.1062632)
Supplement: Supplementary file 10 [file Table2.DOCX]

| **Species/group** | **Gene** | **Protein isoforms** | **note** |
| --- | --- | --- | --- |
| ***Drosophila melanogaster***  Insecta; Holometabola; Diptera; Brachycera; Cyclorrhapha; Drosophilidae | 1 | AAF57110 | 1 protein isoform;  no alternative splicing possible (intronless gene) |
| ***Drosophila grimshawi***  Insecta; Holometabola; Diptera; Brachycera; Cyclorrhapha; Drosophilidae | 1 | GH14346 | 1 protein isoform  (identified in TSA) |
| ***Chymomyza costata***  Insecta; Holometabola; Diptera; Brachycera; Cyclorrhapha; Drosophilidae | 1 | JAECWU010000002 | 1 protein isoform; identified in genome  no alternative splicing possible (intronless gene) |
| ***Ceratitis capitata***  Insecta; Holometabola; Diptera; Brachycera; Cyclorrhapha, Tephritidae | 1 | XP_004523403 | 1 protein isoform; |
| ***Rhagoletis zephyria***  Insecta; Holometabola; Diptera; Brachycera; Cyclorrhapha, Tephritidae | 1 | XP_017479213 | 1 protein isoform  (identified in TSA) |
| ***Zeugodacus cucurbitae***  Insecta; Holometabola; Diptera; Brachycera; Cyclorrhapha, Tephritidae | 1 | XP_011186061 | 1 protein isoform  (identified in TSA) |
| ***Musca domestica***  Insecta; Holometabola; Diptera; Brachycera; Cyclorrhapha, Muscidae | 1 | XP_019890952 | 1 protein isoform; |
| ***Sarcophaga bullata***  Insecta; Holometabola; Diptera; Brachycera; Cyclorrhapha, Sarcophagidae | 1 | TMW47958 | 1 protein isoform  (identified in TSA) |
| ***Heteropsilopus ingenuus***  Insecta; Holometabola; Diptera;  Brachycera; Empidoidea; Dolichopodidae; | 1 | Heteropsilopus ingenuus GCGO01028505 | 1 protein isoform  (identified in TSA) |
| ***Hermetia illucens XP_037913096***  Insecta; Holometabola; Diptera; Brachycera; Stratiomyomorpha; Stratiomyidae | 1 | XP_037913096 | 1 protein isoform  (identified in TSA) |
| ***Machimus arthriticus***  Insecta; Holometabola; Diptera; Brachycera; Asiloidea; Asilidae | 1 | GFZQ01005943 | 1 protein isoform  (identified in TSA) |
| ***Bradysia coprophila***  Insecta; Holometabola; Diptera; Nematocera; Sciaroidea; Sciaridae; | 1 | XP_037029789 | 1 protein isoform  (identified in TSA) |
| ***Phlebotomus kandelakii***  Insecta; Holometabola; Diptera; Nematocera; Psychodoidea; Psychodidae; | 1 | NBJ59035 | 1 protein isoform  (identified in TSA) |
| ***Anopheles albimatus***  Insecta; Holometabola; Diptera; Nematocera; Culicidae | 1 | XP_035774255  XP_035774260 | 2 protein isoforms |
| ***Aedes aegypti***  Insecta; Holometabola; Diptera; Nematocera; Culicidae; | 1 | XP_021703386 | 1 protein isoform |
| ***Culex quinquefasciatus***  Insecta; Holometabola; Diptera; Nematocera; Culicidae | 1 | XP_038108204 | 1 protein isoform |
| ***Ctenocephalides felis***  Insecta; Holometabola; Siphonaptera; Pulicidae | 1 | XP_026472425 | 1 protein isoform |
| ***Boreus hyemalis***  Insecta; Holometabola; Mecoptera | 1 | GAYK02023579 | 1 protein isoform |
| ***Danaus plexippus***  Insecta; Holometabola;  Lepidoptera; Ditrysia;  Nymphalidae | 1 | OWR42709  XP_032518479  XP_032518488 | 3 protein isoforms |
| ***Manduca***  Insecta; Holometabola; Lepidoptera; Ditrysia; Bombycoidea; Sphingidae; | 1 | KAG6442350  XP_030035925 | 2 protein isoforms |
| ***Haliplus fluviatilis***  Insecta; Holometabola;  Coleoptera; Adephaga;  Haliplidae |  | GDMW01032753  GDMW01032754 | 2 protein isoforms |
| ***Onthophagus taurus***  Insecta; Holometabola;  Coleoptera; Polyphaga; Scarabaeidae |  | XP_022916674  XP_022916672  XP_022916673 | 3 protein isoforms |
| ***Tribolium castaneum***  Insecta; Holometabola;  Coleoptera; Polyphaga;  Tenebrionidae |  | XP_015838159  XP_015838158 | 3 protein isoforms |
| ***Xanthostigma xanthostigma***  Insecta; Holometabola;  Raphidioptera; Raphidiidae |  | GAUI02055818 | 1 protein isoform  (identified in TSA) |
| ***Apis mellifera***  Insecta; Holometabola;  Hymenoptera; Apocrita; Aculeata; Apoidea; Apidae |  | XP_006559020  XP_006559021  XP_006559017  XP_026299552  XP_006559019 | 5 protein isoforms |
| ***Nasonia vitripennis***  Insecta; Holometabola;  Hymenoptera; Apocrita; Parasitoidea; Chalcidoidea; Pteromalidae |  | XP_016838712  XP_031781923  XP_031781924  XP_016838713  XP_016838714  XP_031781922 | 6 protein isoforms |
| ***Athalia rosae***  Insecta; Holometabola;  Hymenoptera; Tenthredinoidea; Tenthredinidae |  | XP_020712524  XP_012269380  XP_020712523  XP_020712525  XP_012269382  XP_012269381 | 6 protein isoforms |
| ***Cerobasis guestfalica***  Insecta; Paraneoptera; Psocodea; Psocoptera, Trogiidae |  | GDEA01034316 | 1 protein isoform  (identified in TSA) |
| ***Lachesilla abiesicila***  Insecta; Paraneoptera; Psocodea; Psocoptera, Lachesillidae |  | GDEL01015526 | 1 protein isoform  (identified in TSA) |
| ***Craspedorrhynchus sp.***  Insecta; Paraneoptera; Psocodea; Phthiraptera; Philopteridae |  | GCWN01029268 | 1 protein isoform  (identified in TSA) |
| ***Pediculus humanus***  Insecta; Paraneoptera; Psocodea; Phthiraptera; Pediculidae |  | XP_002427537 | 1 protein isoform |
| ***Frankliniella occidentalis***  Insecta; Paraneoptera;  Thysanoptera; Thripidae |  | XP_026279829  XP_026279830 | 2 protein isoforms |
| ***Thrips palmi***  Insecta; Paraneoptera; Thysanoptera; Thripidae |  | XP_034241879  XP_034241880 | 2 protein isoforms |
| ***Bemisia tabaci***  Insecta; Paraneoptera; Hemiptera; Sternorrhyncha; Aleyrodoidea; Aleyrodidae |  | XP_018907319  XP_018907318  XP_018907320  XP_018907321  XP_018907315  XP_018907316  XP_018907317 | 7 protein isoforms |
| ***Pachypsylla venusta***  Insecta; Paraneoptera; Hemiptera; Sternorrhyncha; Psylloidea; Aphalaridae |  | GAOP01112552 | 1 protein isoforms |
| ***Acyrthosiphon pisum***  Insecta; Paraneoptera; Hemiptera; Sternorrhyncha; Aphidomorpha; Aphididae |  | XP_008186371  XP_001951697  XP_008186372 | 1 protein isoform |
| ***Planococcus citri***  Insecta; Paraneoptera; Hemiptera; Sternorrhyncha; Aphidiformes; Coccoidea; |  | GAXF02008825 | 1 protein isoform  (identified in TSA) |
| ***Okanagana villosa***  Insecta; Hemiptera; Euhemiptera; Cicadoidea; Okanagana |  | GAWQ02049094 | 1 protein isoform  (identified in TSA) |
| ***Rhagovelia antilleana***  Insecta; Paraneoptera; Hemiptera; Heteroptera; Gerromorpha |  | GFOS01047006 | 1 protein isoform |
| ***Rhodnius prolixus***  Insecta; Paraneoptera; Hemiptera; Heteroptera; Cimicomorpha; |  | GECK01038739 | 1 protein isoform |
| ***Cimex lectularius***  Insecta; Paraneoptera; Hemiptera; Heteroptera; Cimicomorpha; |  | XP_014260368  XP_014260727  XP_014260607 | 3 protein isoforms |
| ***Halyomorpha halys***  Insecta; Paraneoptera; Hemiptera; Heteroptera; Pentatomomorpha; |  | XP_014274818  XP_024216977  XP_024216975  XP_024216976 | 4 protein isoforms |
| *Pyrrhocoris apterus*  Insecta; Paraneoptera; Hemiptera; Heteroptera; Pentatomorpha; |  | iso1    OP575299 iso2    OP575300 iso3    OP575301 iso4    OP575302 | 4 protein isoforms |
| ***Apachyus charteceus***  Insecta; Polyneoptera; Dermaptera; |  | GAUW02029097 | 1 protein isoform  (identified in TSA) |
| ***Perlesta teaysia***  Insecta; Polyneoptera; Plecoptera; Perlesta |  | GHRG01076916 | 1 protein isoform  (identified in TSA) |
| ***Locusta migratoria***  Insecta; Polyneoptera; Orthoptera; |  | GETS01026013 | 1 protein isoform  (identified in TSA) |
| ***Timema monikensis***  Insecta; Polyneoptera; Phasmatodea |  | CAD7427940 | 1 protein isoform |
| ***Blattella germanica***  Insecta; Polyneoptera; Blattodea; Blaberoidea; Ectobiidae |  | PSN37932 | 1 protein isoform |
| ***Cryptotermes secundus***  Insecta; Polyneoptera; Blattodea; Blattoidea; Termitoidae; |  | XP_023723334  XP_023723336 | 2 protein isoforms |
| ***Ladona fulva***  Insecta; Pterygota; Palaeoptera; Odonata; Anisoptera; |  | KAG8233907 | 1 protein isoform |
| ***Ecdyonurus insignis***  Insecta; Pterygota; Palaeoptera; Ephemeroptera; Setisura; |  | GCCL01038339 | 1 protein isoform  (identified in TSA) |
| ***Thermobia domestica***  Insecta; Zygentoma; Lepismatidae; |  | GASN02058628 | 1 protein isoform  (identified in TSA) |
| ***Atelura formicaria***  Insecta; Zygentoma; Nicoletiidae; |  | GAYJ02042424 | 1 protein isoform  (identified in TSA) |
| ***Strongylocentrotus purpuratus***  Echinodermata; Echinoidea; Camarodonta; Strongylocentrotidae |  | XP_779963 | 1 protein isoform |
| ***Acanthaster planci***  Echinodermata; Asteroidea; Valvatida; Acanthasteridae |  | XP_022080523  XP_022080521 | 2 protein isoforms |
| ***Saccoglossus kowalevskii***  Hemichordata; Enteropneusta; Harrimaniidae; Saccoglossus |  | XP_006813451  XP_006813452  XP_006813450  XP_006813449  XP_002732288  XP_006813448 | 6 protein isoforms |
| ***Branchiostoma* *floridae***  Chordata; Leptocardii; Branchiostomidae |  | XP_035696214 | 1 protein isoform |
| ***Petromyzon marinus***  Metazoa; Chordata; Vertebrata; Cyclostomata; Hyperoartia; | CKI  LOC116955997 | XP_032833279 | 1 protein isoform |
|  | CKI-like  LOC116949661 | XP_032823110  XP_032823111 | 2 protein isoforms |
| ***Danio rerio***  Chordata; Vertebrata; Actinopteri; Teleostei; Cypriniformes; | CKI delta-a | NP_955877 | 1 protein isoform |
|  | CKI delta-b | NP_998415 | 1 protein isoform |
|  | CKI epsilon | NP_997912 | 1 protein isoform |
| ***Xenopus laevis***  Chordata; Vertebrata; Tetrapoda; Amphibia; Anura; | CKI delta-S | XP_018096015 | 1 protein isoform |
|  | CKI delta-L | XP_018090607  XP_018090608  XP_018090609 | 3 protein isoforms |
|  | CKI epsilon | XP_018112072 | 1 protein isoform |
| ***Anolis carolinensis***  Chordata; Vertebrata; Tetrapoda; Sauria; Lepidosauria; Squamata; | CKI delta | XP_003217335  XP_008102766  XP_008102767 | 3 protein isoforms |
|  | CKI epsilon | XP_003227197 | 1 protein isoform |
| ***Gallus gallus***  Chordata; Vertebrata; Archosauria; Dinosauria; Aves; Neognathae; | CKI delta | XP_415634  XP_040505424 | 2 protein isoforms |
|  | CKI epsilon | NP_989708 | 1 protein isoform |
| ***Vombatus ursinus***  Chordata; Vertebrata; Tetrapoda; Mammalia; Diprotodontia; | CKI delta | XP_027728943  XP_027728944  XP_027728945 | 3 protein isoforms |
|  | CKI epsilon | XP_027727465  XP_027727484  XP_027727496 | 3 protein isoforms |
| ***Mus musculus***  Chordata; Vertebrata; Tetrapoda; Mammalia; Rodentia; | CKI delta | NP_620690  NP_082150 | 2 protein isoforms |
|  | CKI epsilon | NP_001276828  NP_038795 | 2 protein isoforms |
| ***Homo sapiens***  Chordata; Vertebrata; Tetrapoda; Mammalia; Primates; | CKI delta | NP_001884  NP_620693  NP_001350678 | 3 protein isoforms |
|  | CKI epsilon | NP_689407 | 1 protein isoform |
