# Supplementary material for: Evolution of casein kinase 1 and functional analysis of new doubletime mutants in Drosophila
Source: Front Physiol. 2022 Dec 14;13:1062632. doi: 10.3389/fphys.2022.1062632 (PMC9794997; doi:10.3389/fphys.2022.1062632)
Supplement: Supplementary file 11 [file Table5.DOCX]

## Supplementary text describing circadian clock gene terminology

***Dosophila-type timeless* (*d*-*tim*), dTIMELESS (dTIM), (CG3234)**

is used for the *Drosophila*-type *timeless* gene/transcript (in italics) and protein (in capitals) in this publication. However, the terminology varied in history:

Simple abbreviations ***tim*** and **TIM** were used in the early studies (Ashmore et al., 2003; Myers et al 1995; Sehgal et al., 1994; Stanewsky et al., 2002; Hunter-Ensor et al 1996; and many others). With the discovery of an alternative transcription start, ***ls-tim***, ***s-tim*** and **L-TIM**, **S-TIM** were introduced (Rosato et al., 1997; Tauber et al., 2007; with Peschel et al., 2009, using L‑Tim, S-Tim and Tim for protein). Additional splicing variants were described and labeled ***tim‑sc***, ***tim‑M***, ***tim-cold***, and ***tim-L*** (Martin Andagua et al., 2019, Boothroyd et al. 2007; Foley et al. 2017). In mammals, a homologous gene *timeless* (*tim*) was discovered in the late 90s (Zylka et al., 1998). However, a few years later it turned out that *Drosophila* and other insect species also possess a homolog of this mammalian ***tim.*** Thus, *Drosophila* has two *timeless* genes (two paralogs): the *Drosophila* type and the mammalian type. The first (CG3234) was sometimes labeled as ***tim1*** (**TIM1** for the protein) and the latter as ***tim2*** (**TIM2**) (Benna et al. 2000, 2010; CG7855). However, Reppert’s laboratory published a parallel terminology with **TIM** for the *Drosophila*-type and **TIMEOUT** for the mammalian type in *Drosophila*, **mTIM** for the mouse protein, and ***C.e.* TIM-1** for protein from *Caenorhabditis elegans* (Gotter et al., 2000). The latter one is rather unfortunate because the *C. elegans* protein is actually branching with the mammalian types (labeled as TIM2 by Benna). Later, Engelen used **dTIM** and **dTIM‑2 or dTIM2** for the two *Drosophila* paralogs; **s-TIM** and **l-TIM** for alternative transcription starts, whereas **TIM** served as abbreviation for the mammalian protein (Engelen et al., 2013). In 2017, Bazalova et al. published a comparative study between fruit fly and house fly in which the *Drosophila*-type *timeless* was analyzed and described *Drosophila tim* as ***Dm_tim*** and *Musca domestica* as ***Md_tim***. A recent study, highlighting the loss of several circadian genes across bilateria and insects, used TIMELESS-d (TIM-d) for light sensitive TIM (or TIM1) and TIMELESS-m (TIM-m) for light-insensitive and mammalian-type TIM (or TIM2, timeout) (Kotwica-Rolinska et al., 2022).

***Dosophila-type cryptochrome* (*d-cry*), dCRYPTOCHROME (dCRY)** is used for the *Drosophila*-type *cryptochrome* gene/transcript (in italics) and protein (in capitals) in this publication. Several studies also used **CRY1** for the *Drosophila*-type and **CRY2** for the mammalian-type, although the latter is also present in some but not all insects (Zhu et al., 2006; Yuan et al., 2007). Importantly, mammals have two CRYPTOCHROME paralogs, historically labeled as **CRY1** and **CRY2**, yet both belong to the mammalian type. Furthermore, the name ‘evolved’ due to functional variance between mammalian and *Drosophila* CRY: the latter often referred to as dCRY (Vaidya et al., 2013; Baik et. al. 2019; Chandrasekaran et al., 2021 and others) and mammalian CRY referred to as mCRY1 and mCRY2 (e.g., Oster et al., 2003; Collins et al.,2006). Recent biochemical and the structural studies of *Drosophila* CRYPTOCHROME have been using different alternative versions such as DmCry (Arthaut et al., 2017), *Dm*Cry (Berntsson et al., 2019; Einholz et al., 2021) and DmCRY (Kutta et al., 2018). Furthermore, some of the earlier studies used *dcry* or *DCry* for the gene, and DCRY for the protein, respectively (Ishikawa et al., 1999 Egan et al., 1999). A recent phylogenetic study adapts the terminology such as PCRY (plant-specific), ACRY (cnidirian-specific), DCRY (*Drosophila*-type) and MCRY (mammalian-type), all used for protein (Deppisch et al., 2022). Studies on non-model insects reported the presence of CRY which is sequentially and functionally more conserved to the mammalian version of CRY, hence distinguishing both as *Drosophila*-type CRYPTOCHROME (CRY-d) and mammalian-type CRYPTOCHROME (CRY-m) (Rubin et al., 2006; Ikeno et al., 2008, 2011a,b; Ingram et al., 2012, Kotwica-Rolinska et al., 2022).

***doubletime* (*dbt*)**, **DOUBLETIME** was discovered as a circadian clock protein in 1998 in *Drosophila* (Price et al., 1998; Kloss et al., 1998). One year later, it turned out that the *dbt* gene is identical to ***disc overgrown*, *dco*,** a gene that was identified for its developmental role (Zilian et al., 1999). Mammalian literature discriminates between **α**, **γ**, **ε**, and **δ** isoforms of the casein kinase I proteins (**CKI** or **CK1**), even though each of them is encoded by a completely different gene (and there are even three paralogous γ genes in mammals) (Zhang et al., 1996; Green and Bennett, 1998; Gross and Anderson, 1998; Rivers et al., 1998; Knippschild et al., 2005; Zheng et al., 2014).

## Supplementary references:

Anduaga, A. M., Evantal, N., Patop, I. L., Bartok, O., Weiss, R., & Kadener, S. (2019). Thermosensitive alternative splicing senses and mediates temperature adaptation in Drosophila. *Elife*, *8*, e44642. doi: 10.7554/eLife.44642

Arthaut, L. D., Jourdan, N., Mteyrek, A., Procopio, M., El-Esawi, M., d’Harlingue, A., ... & Ahmad, M. (2017). Blue-light induced accumulation of reactive oxygen species is a consequence of the Drosophila cryptochrome photocycle. *PloS one*, *12*(3), e0171836. doi: 10.1371/journal.pone.0171836

Ashmore, L. J., Sathyanarayanan, S., Silvestre, D. W., Emerson, M. M., Schotland, P., and Sehgal, A. (2003). Novel insights into the regulation of the timeless protein. *J. Neurosci.* 23, 7810–7819. doi: 10.1523/jneurosci.23-21-07810.2003

Baik, L. S., Au, D. D., Nave, C., Foden, A. J., Enrriquez-Villalva, W. K., & Holmes, T. C. (2019). Distinct mechanisms of Drosophila CRYPTOCHROME-mediated light-evoked membrane depolarization and in vivo clock resetting. *Proceedings of the National Academy of Sciences*, *116*(46), 23339-23344. doi: 10.1073/pnas.1905023116

Bazalova, O., and Dolezel, D. (2017). Daily ktivity of the housefly, Musca domestica, is influenced by temperature independent of 3’ UTR period gene splicing. *G3-Genes Genomes Genet.* 7, 2637–2649. doi: 10.1534/g3.117.042374

Benna, C., Scannapieco, P., Piccin, A., Sandrelli, F., Zordan, M., Rosato, E., … & Costa, R. (2000). A second timeless gene in Drosophila shares greater sequence similarity with mammalian tim. *Current biology*, *10*(14), R512-R513. doi: 10.1016/S0960-9822(00)00594-7

Benna, C., Bonaccorsi, S., Wülbeck, C., Helfrich-Förster, C., Gatti, M., Kyriacou, C. P., … & Sandrelli, F. (2010). Drosophila timeless2 is required for chromosome stability and circadian photoreception. *Current Biology*, *20*(4), 346-352. doi: 10.1016/j.cub.2009.12.

Berntsson, O., Rodriguez, R., Henry, L., Panman, M. R., & Hughes, A. J. Photoactivation of drosophila melanogaster cryptochrome through sequential conformational transitions. Sci. Adv. 5 (7), eaaw1531 (2019). doi: 10.1126/sciadv.aaw1531

Boothroyd, C.E., Wijnen, H., Naef, F., Saez, L., Young, M.W. (2007). Integration of light and temperature in the regulation of circadian gene expression in Drosophila.  *PLoS Genetics*. 3(4): e54. doi: 10.1371/journal.pgen.0030054

Chandrasekaran, S., Schneps, C. M., Dunleavy, R., Lin, C., DeOliveira, C. C., Ganguly, A., & Crane, B. R. (2021). Tuning flavin environment to detect and control light-induced conformational switching in Drosophila cryptochrome. *Communications biology*, *4*(1), 1-12. doi: 10.1038/s42003-021-01766-2.

Collins, B., Mazzoni, E. O., Stanewsky, R., & Blau, J. (2006). Drosophila CRYPTOCHROME is a circadian transcriptional repressor. *Current Biology*, *16*(5), 441-449. doi:10.1016/j.cub.2006.01.034

Deppisch, P., Helfrich-Förster, C., & Senthilan, P. R. (2022). The Gain and Loss of Cryptochrome/Photolyase Family Members during Evolution. *Genes*, *13*(9), 1613. doi: 10.3390/genes13091613

Egan, E. S., Franklin, T. M., Hilderbrand-Chae, M. J., McNeil, G. P., Roberts, M. A., Schroeder, A. J., ... & Jackson, F. R. (1999). An extraretinally expressed insect cryptochrome with similarity to the blue light photoreceptors of mammals and plants. *Journal of Neuroscience*, *19*(10), 3665-3673. doi: 10.1523/JNEUROSCI.19-10-03665.1999

Einholz, C., Nohr, D., Rodriguez, R., Topitsch, A., Kern, M., Goldmann, J., ... & Schleicher, E. (2021). pH-dependence of signaling-state formation in Drosophila cryptochrome. *Archives of Biochemistry and Biophysics*, *700*, 108787. doi: 10.1016/j.abb.2021.108787

Engelen, E., Janssens, R. C., Yagita, K., Smits, V. A., van der Horst, G. T., & Tamanini, F. (2013). Mammalian TIMELESS is involved in period determination and DNA damage-dependent phase advancing of the circadian clock. *PloS one*, *8*(2), e56623. doi: 10.1371/journal.pone.0056623

Foley, L. E., Ling J., Jsohi R., Evantal N. Kadener S., & Emery P, (2017). *Drosophila* PSI controls circadian period and the phase of circadian behavior under temperature cycle via *tim* splicing. *eLIFE* 8;8:e50063. doi: 10.7554/eLife.50063.

Gotter, A. L., Manganaro, T., Weaver, D. R., Kolakowski, L. F., Possidente, B., Sriram, S., … & Reppert, S. M. (2000). A time-less function for mouse timeless. *Nature neuroscience*, *3*(8), 755-756. doi: 10.1038/77653

Green, C. L., & Bennett, G. S. (1998). Identification of four alternatively spliced isoforms of chicken casein kinase I alpha that are all expressed in diverse cell types. *Gene*, *216*(1), 189-195. doi: 10.1016/s0378-1119(98)00291-1.

Gross, S. D., & Anderson, R. A. (1998). Casein kinase I: spatial organization and positioning of a multifunctional protein kinase family. *Cellular signalling*, *10*(10), 699-711. doi: 10.1016/s0898-6568(98)00042-4.

Hunter-Ensor, M., Ousley, A., & Sehgal, A. (1996). Regulation of the Drosophila protein timeless suggests a mechanism for resetting the circadian clock by light. *Cell*. 84(5), 677-685. doi: 10.1016/s0092-8674(00)81046-6

Ikeno, T., Numata, H., & Goto, S. G. (2008). Molecular characterization of the circadian clock genes in the bean bug, Riptortus pedestris, and their expression patterns under long-and short-day conditions. *Gene*, *419*(1-2), 56-61. doi: 10.1016/j.gene.2008.05.002.

Ikeno, T., Katagiri, C., Numata, H., & Goto, S. G. (2011a). Causal involvement of mammalian‐type cryptochrome in the circadian cuticle deposition rhythm in the bean bug Riptortus pedestris. *Insect molecular biology*, *20*(3), 409-415. doi: 10.1111/j.1365-2583.2011.01075.x.

Ikeno, T., Numata, H., & Goto, S. G. (2011b). Photoperiodic response requires mammalian-type cryptochrome in the bean bug Riptortus pedestris. *Biochemical and biophysical research communications*, *410*(3), 394-397. doi: 10.1016/j.bbrc.2011.05.142.

Ingram, K. K., Kutowoi, A., Wurm, Y., Shoemaker, D., Meier, R., & Bloch, G. (2012). The molecular clockwork of the fire ant Solenopsis invicta. *PloS one*, *7*(11), e45715. doi: 10.1371/journal.pone.0045715.

Ishikawa, T., Matsumoto, A., Kato Jr, T., Togashi, S., Ryo, H., Ikenaga, M., ... & Tanimura, T. (1999). DCRY is a Drosophila photoreceptor protein implicated in light entrainment of circadian rhythm. *Genes to Cells*, *4*(1), 57-65. doi: 10.1046/j.1365-2443.1999.00237.x

Kloss, B., Price, J. L., Saez, L., Blau, J., Rothenfluh, A., Wesley, C. S., & Young, M. W. (1998). The Drosophila clock gene double-time encodes a protein closely related to human casein kinase Iε. *Cell*, *94*(1), 97-107. doi: 10.1016/S0092-8674(00)81225-8

Knippschild, U., Gocht, A., Wolff, S., Huber, N., Löhler, J., and Stöter, M. (2005). The casein kinase 1 family: Participation in multiple cellular processes in eukaryotes. *Cell Signal* 17, 675–689. doi: 10.1016/j.cellsig.2004.12.011.

Kotwica-Rolinska, J., Chodakova, L., Smykal, V., Damulewicz, M., Provaznik, J., et al. (2022a). Loss of Timeless Underlies an Evolutionary Transition within the Circadian Clock. *Mol. Biol. Evol.* 39, doi: 10.1093/molbev/msab346

Kutta, R. J., Archipowa, N., & Scrutton, N. S. (2018). The sacrificial inactivation of the blue-light photosensor cryptochrome from Drosophila melanogaster. *Physical Chemistry Chemical Physics*, *20*(45), 28767-28776. doi: 10.1039/c8cp04671a

Myers, M. P., Wager-Smith, K., Wesley, C. S., Young, M. W., & Sehgal, A. (1995). Positional cloning and sequence analysis of the Drosophila clock gene, timeless. *Science*, *270*(5237), 805-808. doi: 10.1126/science.270.5237.805

Oster, H., Baeriswyl, S., Van Der Horst, G. T., & Albrecht, U. (2003). Loss of circadian rhythmicity in aging mPer1-/-mCry2-/-mutant mice. *Genes & Development*, *17*(11), 1366-1379. doi: 10.1101/gad.256103.

Peschel, N., Chen, K. F., Szabo, G., Stanewsky, R. (2009). Light-Dependent Interactions between the Drosophila Circadian Clock Factors Cryptochrome, Jetlag, and Timeless. *Current Biology* 19(3): 241- 247. doi: 10.1016/j.cub.2008.12.042

Price, J. L., Blau, J., Rothenfluh, A., Abodeely, M., Kloss, B., & Young, M. W. (1998). double-time is a novel Drosophila clock gene that regulates PERIOD protein accumulation. *Cell*, *94*(1), 83-95. doi: 10.1016/S0092-8674(00)81224-6

Rivers, A., Gietzen, K. F., Vielhaber, E., & Virshup, D. M. (1998). Regulation of Casein Kinase I ε and Casein Kinase I δ by anin Vivo Futile Phosphorylation Cycle. *Journal of Biological Chemistry*, *273*(26), 15980-15984. doi: 10.1074/jbc.273.26.15980.

Rosato, E., Trevisan, A., Sandrelli, F., Zordan, M., Kyriacou, C. P., & Costa, R. (1997). Conceptual translation of timeless reveals alternative initiating methionines in Drosophila. *Nucleic acids research*, *25*(3), 455-457. doi: 10.1093/nar/25.3.455.

Rubin, E. B., Shemesh, Y., Cohen, M., Elgavish, S., Robertson, H. M., & Bloch, G. (2006). Molecular and phylogenetic analyses reveal mammalian-like clockwork in the honey bee (Apis mellifera) and shed new light on the molecular evolution of the circadian clock. *Genome research*, *16*(11), 1352-1365. doi: 10.1101/gr.5094806.

Sehgal, A., Price, J. L., Man, B., and Young, M. W. (1994). Loss of circadian behavioral rhythms and per RNA oscillations in the Drosophila mutant timeless. *Science* 263, 1603–1606. doi: 10.1126/science.8128246

Stanewsky, R., Lynch, K. S., Brandes, C., & Hall, J. C. (2002). Mapping of elements involved in regulating normal temporal period and timeless RNA expression patterns in Drosophila melanogaster. *Journal of biological rhythms*, *17*(4), 293-306. doi: 10.1177/074873002129002609

Tauber, E., Zordan, M., Sandrelli, F., Pegoraro, M., Osterwalder, N., Breda, C., … & Costa, R. (2007). Natural selection favors a newly derived timeless allele in Drosophila melanogaster. *Science*, *316*(5833), 1895-1898. doi: 10.1126/science.1138412

Vaidya, A. T., Top, D., Manahan, C. C., Tokuda, J. M., Zhang, S., Pollack, L., ... & Crane, B. R. (2013). Flavin reduction activates Drosophila cryptochrome. *Proceedings of the National Academy of Sciences*, *110*(51), 20455-20460. doi: 10.1073/pnas.1313336110.

Yuan, Q., Metterville, D., Briscoe, A. D., & Reppert, S. M. (2007). Insect cryptochromes: gene duplication and loss define diverse ways to construct insect circadian clocks. *Molecular biology and evolution*, *24*(4), 948-955. doi: 10.1093/molbev/msm011

Zhang, J., Gross, S. D., Schroeder, M. D., & Anderson, R. A. (1996). Casein kinase I α and αL: alternative splicing-generated kinases exhibit different catalytic properties. *Biochemistry*, *35*(50), 16319-16327. doi: 10.1021/bi9614444.

Zheng, X., Sowcik, M., Chen, D., and Sehgal, A. (2014). Casein Kinase 1 Promotes Synchrony of the Circadian Clock Network. *Mol Cell Biol* 34, 2682–2694. doi: 10.1128/mcb.01571-13.

Zhu, H., Yuan, Q., Briscoe, A. D., Froy, O., Casselman, A., & Reppert, S. M. (2006). The two CRYs of the butterfly. *Current Biology*, *16*(7), 730. doi: 10.1016/j.cub.2006.03.02

Zilian, O., Frei, E., Burke, R., Brentrup, D., Gutjahr, T., Bryant, P. J., & Noll, M. (1999). double-time is identical to discs overgrown, which is required for cell survival, proliferation and growth arrest in Drosophila imaginal discs. *Development*, *126*(23), 5409-5420. doi: 10.1242/dev.126.23.5409

Zylka, M. J., Shearman, L. P., Levine, J. D., Jin, X., Weaver, D. R., & Reppert, S. M. (1998). Molecular analysis of mammalian timeless. *Neuron*, *21*(5), 1115-1122. doi: 10.1016/S0896-6273(00)80628-5
