# Supplementary material for: Evolution of casein kinase 1 and functional analysis of new doubletime mutants in Drosophila
Source: Front Physiol. 2022 Dec 14;13:1062632. doi: 10.3389/fphys.2022.1062632 (PMC9794997; doi:10.3389/fphys.2022.1062632)
Supplement: Supplementary file 12 [file Table3.DOCX]

| Primer name | Sequence | Used for |
| --- | --- | --- |
| dbtBMHRF | Gatggtcgactctagaagtgatcgcgagacgggt | Outside primer for amplifying 5' homology region |
| dbtBMHRR | gatggtcgacaagcttGTATGGATTGGATTCGCTAGAGC | Outside primer for amplifying 3' homology region |
| dbtPamT1mutF | CGCCTTGCCATGGCAAGGCTTAAAGGCAGCCAACA | Introduces Pam site mutation for gRNA target |
| dbtPamT1mutR | TTGCCATGGCAAGGCGCCCAGATTGAAGTACA | Introduces Pam site mutation for gRNA target |
| dbtSalIF | GAGAAGAAGCTGTCGACCTCGATTGTGGTGCTG | Introduces SalI site |
| dbtSalIR | GTCGACAGCTTCTTCTCCGAGATCCTCT | Introduces SalI site |
| dbtK224DF | CCAACAAGAGGCAAGACTACGAGAGGATCTCGGAGAA | Introduces K224D mutation |
| dbtK224DR | GTCTTGCCTCTTGTTGGCTGCCTTTAAGCC | Introduces K224D mutation |
| dbtK224EF | CCAACAAGAGGCAAGAGTACGAGAGGATCTCGGAGAA | Introduces K224E mutation |
| dbtK224ER | CTCTTGCCTCTTGTTGGCTGCCTTTAAGCC | Introduces K224E mutation |
| SeqDbtF | CATCGCGACATCAAGCCGGATAAC | Sequences modified dbt region |
| SeqDbtR | TGGAGTTGCTGTTGCGCTGCACTGCC | Sequences modified dbt region |
| dbtscreenF1 | CATCGCGACATCAAGCCGGATAACTTCC | Molecular screen of transformants via SalI site |
| dbtscreenR | TGCCGCATCCGTATCGAAGGCCGACGC | Molecular screen of transformants via SalI site |
| DbtgRNAT1F | GTCGGTGGCAGTAATCGGGACGC | gRNA target |
| DbtgRNAT1R | AAACGCGTCCCGATTACTGCCACC | gRNA target |
