# Supplementary material for: Evolution of casein kinase 1 and functional analysis of new doubletime mutants in Drosophila
Source: Front Physiol. 2022 Dec 14;13:1062632. doi: 10.3389/fphys.2022.1062632 (PMC9794997; doi:10.3389/fphys.2022.1062632)
Supplement: Supplementary file 13 [file Table4.DOCX]

| Number of families | 1 |  |  |  |  |  |  |  |
| --- | --- | --- | --- | --- | --- | --- | --- | --- |
| Number of comparisons per family | 3 |  |  |  |  |  |  |  |
| Alpha | 0.05 |  |  |  |  |  |  |  |
|  |  |  |  |  |  |  |  |  |
| Dunnett's multiple comparisons test | Mean Diff. | 95.00% CI of diff. | Below threshold? | Summary | Adjusted P Value |  |  |  |
| CS vs. DBT ?370-440 | -0.6236 | -0.9589 to -0.2883 | Yes | *** | 0.0008 |  |  |  |
| CS vs. DBT ?366-370 | 0.6424 | 0.3134 to 0.9714 | Yes | *** | 0.0006 |  |  |  |
| CS vs. DBT ?411-440 | 0.3824 | 0.04650 to 0.7182 | Yes | * | 0.0256 |  |  |  |
|  |  |  |  |  |  |  |  |  |
| Test details | Mean 1 | Mean 2 | Mean Diff. | SE of diff. | N1 | N2 | q | DF |
| CS vs. DBT ?370-440 | 23.60 | 24.23 | -0.6236 | 0.1244 | 58 | 45 | 5.014 | 12 |
| CS vs. DBT ?366-370 | 23.60 | 22.96 | 0.6424 | 0.1221 | 58 | 55 | 5.263 | 12 |
| CS vs. DBT ?411-440 | 23.60 | 23.22 | 0.3824 | 0.1246 | 58 | 44 | 3.069 | 12 |

| Number of families | 1 |  |  |  |  |  |  |  |
| --- | --- | --- | --- | --- | --- | --- | --- | --- |
| Number of comparisons per family | 2 |  |  |  |  |  |  |  |
| Alpha | 0.05 |  |  |  |  |  |  |  |
|  |  |  |  |  |  |  |  |  |
| Dunnett's multiple comparisons test | Mean Diff. | 95.00% CI of diff. | Below threshold? | Summary | Adjusted P Value |  |  |  |
| y w vs. K224D | 3.011 | 1.693 to 4.329 | Yes | ** | 0.0011 |  |  |  |
| y w vs. K224E | 4.656 | 3.343 to 5.968 | Yes | *** | 0.0001 |  |  |  |
|  |  |  |  |  |  |  |  |  |
| Test details | Mean 1 | Mean 2 | Mean Diff. | SE of diff. | N1 | N2 | q | DF |
| y w vs. K224D | 23.82 | 20.81 | 3.011 | 0.4673 | 40 | 60 | 6.445 | 6 |
| y w vs. K224E | 23.82 | 19.17 | 4.656 | 0.4653 | 40 | 91 | 10.01 | 6 |
