# Supplementary material for: Evolution of casein kinase 1 and functional analysis of new doubletime mutants in Drosophila
Source: Front Physiol. 2022 Dec 14;13:1062632. doi: 10.3389/fphys.2022.1062632 (PMC9794997; doi:10.3389/fphys.2022.1062632)
Supplement: Supplementary file 17 [file Image1.PDF]

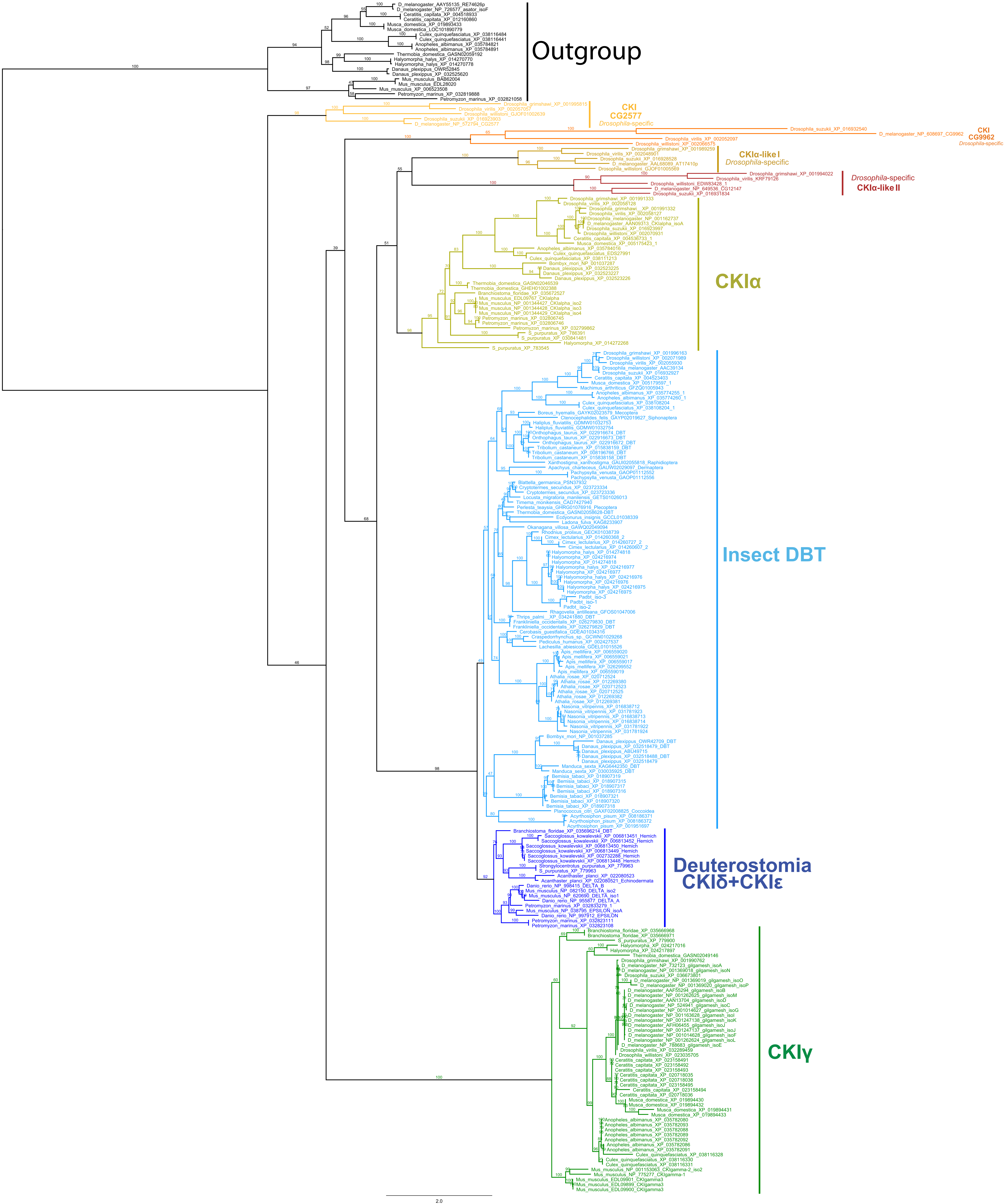
Outgroup
D_melanogaster_AAY55135_RE74626p
D_melanogaster_NP_726577_asator_isoF
Ceratitis_capitata_XP_004518933
Ceratitis_capitata_XP_012160860
Musca_domestica_XP_019893433
Musca_domestica_LOC101890779
Culex_quinquefasciatus_XP_038116484
Culex_quinquefasciatus_XP_038116441
Anopheles_albimanus_XP_035784821
Anopheles_albimanus_XP_035784891
Thermobia_domestica_GASN02059192
Halyomorpha_halys_XP_014270770
Halyomorpha_halys_XP_014270778
Danaus_plexippus_OWR52845
Danaus_plexippus_XP_032525620
Mus_musculus_BAB62004
Mus_musculus_EDL28020
Mus_musculus_XP_006523508
Petromyzon_marinus_XP_032819888
Petromyzon_marinus_XP_032821058
CKI CG2577
Drosophila-specific
Drosophila_grimshawi_XP_001995815
Drosophila_virilis_XP_002057057
Drosophila_willistoni_GJOF01002639
Drosophila_suzukii_XP_016923903
D_melanogaster_NP_572794_CG2577
CKI CG9962
Drosophila-specific
Drosophila_suzukii_XP_016932540
D_melanogaster_NP_608697_CG9962
Drosophila_virilis_XP_002052097
Drosophila_willistoni_XP_002066575
CKIα-like I
Drosophila-specific
Drosophila_grimshawi_XP_001989259
Drosophila_virilis_XP_002048901
Drosophila_suzukii_XP_016928528
D_melanogaster_AAL68089_AT17410p
Drosophila_willistoni_GJOF01005569
Drosophila-specific
CKIα-like II
Drosophila_grimshawi_XP_001994022
Drosophila_virilis_KRF79126
Drosophila_willistoni_EDW83428_1
D_melanogaster_NP_649536_CG12147
Drosophila_suzukii_XP_016931834
CKIα
Drosophila_grimshawi_XP_001991333
Drosophila_virilis_XP_002058128
Drosophila_melanogaster_NP_001162737
D_melanogaster_AAN09313_CKIalpha_isoA
Drosophila_suzukii_XP_016923997
Drosophila_willistoni_XP_002070931
Ceratitis_capitata_XP_004536733_1
Musca_domestica_XP_005175423_1
Anopheles_albimanus_XP_035784016
Culex_quinquefasciatus_EDS27991
Culex_quinquefasciatus_XP_038111213
Bombyx_mori_NP_001037287
Danaus_plexippus_XP_032523225
Danaus_plexippus_XP_032523227
Danaus_plexippus_XP_032523226
Thermobia_domestica_GASN02046539
Thermobia_domestica_GHEH01002388
Branchiostoma_floridae_XP_035672527
Mus_musculus_EDL09767_CKIalpha
Mus_musculus_NP_001344427_CKIalpha_iso2
Mus_musculus_NP_001344428_CKIalpha_iso3
Mus_musculus_NP_001344429_CKIalpha_iso4
Petromyzon_marinus_XP_032806745
Petromyzon_marinus_XP_032806746
Petromyzon_marinus_XP_032799862
S_purpuratus_XP_786391
S_purpuratus_XP_030841481
Halyomorpha_XP_014272268
S_purpuratus_XP_783545
Insect DBT
Drosophila_grimshawi_XP_001996163
Drosophila_willistoni_XP_002071989
Drosophila_virilis_XP_002055930
Drosophila_melanogaster_AAC39134
Drosophila_suzukii_XP_016932927
Ceratitis_capitata_XP_004523403
Musca_domestica_XP_005179597_1
Machimus_arthriticus_GFZQ01005943
Anopheles_albimanus_XP_035774255_1
Anopheles_albimanus_XP_035774260_1
Culex_quinquefasciatus_XP_038108204
Culex_quinquefasciatus_XP_038108204_1
Boreus_hyemalis_GAYK02023579_Mecoptera
Ctenocephalides_felis_GAYP02019627_Siphonaptera
Haliplus_fluviatilis_GDMW01032753
Haliplus_fluviatilis_GDMW01032754
Onthophagus_taurus_XP_022916674_DBT
Onthophagus_taurus_XP_022916673_DBT
Onthophagus_taurus_XP_022916672_DBT
Tribolium_castaneum_XP_015838159_DBT
Tribolium_castaneum_XP_008196766_DBT
Tribolium_castaneum_XP_015838158_DBT
Xanthostigma_xanthostigma_GAUI02055818_Raphidioptera
Apachyus_charteceus_GAUW02029097_Dermaptera
Pachypsylla_venusta_GAOP01112552
Pachypsylla_venusta_GAOP01112556
Blattella_germanica_PSN37932
Cryptotermes_secundus_XP_023723334
Cryptotermes_secundus_XP_023723336
Locusta_migratoria_manilensis_GETS01026013
Timema_monikensis_CAD7427940
Perlesta_teaysia_GHRG01076916_Plecoptera
Thermobia_domestica_GASN02058628-DBT
Ecdyonurus_insignis_GCCL01038339
Ladona_fulva_KAG8233907
Okanagana_villosa_GAWQ02049094
Rhodnius_prolixus_GECK01038739
Cimex_lectularius_XP_014260368_2
Cimex_lectularius_XP_014260727_2
Cimex_lectularius_XP_014260607_2
Halyomorpha_halys_XP_014274818
Halyomorpha_XP_024216974
Halyomorpha_XP_014274818
Halyomorpha_halys_XP_024216977
Halyomorpha_XP_024216977
Halyomorpha_halys_XP_024216976
Halyomorpha_XP_024216976
Halyomorpha_halys_XP_024216975
Halyomorpha_XP_024216975
Padbt_iso-3
Padbt_iso-1
Padbt_iso-2
Rhagovelia_antilleana_GFOS01047006
Thrips_palmi_XP_034241880_DBT
Frankliniella_occidentalis_XP_026279830_DBT
Frankliniella_occidentalis_XP_026279829_DBT
Cerobasis_guestfalica_GDEA01034316
Craspedorrhynchus_sp._GCWN01029268
Pediculus_humanus_XP_002427537
Lachesilla_abiesicola_GDEL01015526
Apis_mellifera_XP_006559020
Apis_mellifera_XP_006559021
Apis_mellifera_XP_006559017
Apis_mellifera_XP_026299552
Apis_mellifera_XP_006559019
Athalia_rosae_XP_020712524
Athalia_rosae_XP_012269380
Athalia_rosae_XP_020712523
Athalia_rosae_XP_020712525
Athalia_rosae_XP_012269382
Athalia_rosae_XP_012269381
Nasonia_vitripennis_XP_016838712
Nasonia_vitripennis_XP_031781923
Nasonia_vitripennis_XP_016838713
Nasonia_vitripennis_XP_016838714
Nasonia_vitripennis_XP_031781922
Nasonia_vitripennis_XP_031781924
Bombyx_mori_NP_001037285
Danaus_plexippus_OWR42709_DBT
Danaus_plexippus_XP_032518479_DBT
Danaus_plexippus_ABU49715
Danaus_plexippus_XP_032518488_DBT
Danaus_plexippus_XP_032518479
Manduca_sexta_KAG6442350_DBT
Manduca_sexta_XP_030035925_DBT
Bemisia_tabaci_XP_018907319
Bemisia_tabaci_XP_018907315
Bemisia_tabaci_XP_018907317
Bemisia_tabaci_XP_018907316
Bemisia_tabaci_XP_018907321
Bemisia_tabaci_XP_018907320
Bemisia_tabaci_XP_018907318
Planococcus_citri_GAXF02008825_Coccoidea
Acyrthosiphon_pisum_XP_008186371
Acyrthosiphon_pisum_XP_008186372
Acyrthosiphon_pisum_XP_001951697
Deuterostomia
CKIδ+CKIε
Branchiostoma_floridae_XP_035696214_DBT
Saccoglossus_kowalevskii_XP_006813451_Hemich
Saccoglossus_kowalevskii_XP_006813452_Hemich
Saccoglossus_kowalevskii_XP_006813450_Hemich
Saccoglossus_kowalevskii_XP_006813449_Hemich
Saccoglossus_kowalevskii_XP_002732288_Hemich
Saccoglossus_kowalevskii_XP_006813448_Hemich
Strongylocentrotus_purpuratus_XP_779963
S_purpuratus_XP_779963
Acanthaster_planci_XP_022080523
Acanthaster_planci_XP_022080521_Echinodermata
Danio_rerio_NP_998415_DELTA_B
Mus_musculus_NP_082150_DELTA_iso2
Mus_musculus_NP_620690_DELTA_iso1
Danio_rerio_NP_955877_DELTA_A
Petromyzon_marinus_XP_032833279_1
Mus_musculus_NP_038795_EPSILON_isoA
Danio_rerio_NP_997912_EPSILON
Petromyzon_marinus_XP_032823111
Petromyzon_marinus_XP_032823108
CKIγ
Branchiostoma_floridae_XP_035666968
Branchiostoma_floridae_XP_035666971
S_purpuratus_XP_779900
Halyomorpha_XP_024217016
Halyomorpha_XP_024217897
Thermobia_domestica_GASN02049146
Drosophila_grimshawi_XP_001990762
D_melanogaster_NP_732123_gilgamesh_isoA
D_melanogaster_NP_001369018_gilgamesh_isoN
Drosophila_suzukii_XP_036673801
D_melanogaster_NP_001369019_gilgamesh_isoO
D_melanogaster_NP_001369020_gilgamesh_isoP
D_melanogaster_AAF55294_gilgamesh_isoB
D_melanogaster_NP_001262625_gilgamesh_isoM
D_melanogaster_AAN13704_gilgamesh_isoD
D_melanogaster_NP_524941_gilgamesh_isoC
D_melanogaster_NP_001014627_gilgamesh_isoG
D_melanogaster_NP_001163628_gilgamesh_isoI
D_melanogaster_NP_001247138_gilgamesh_isoK
D_melanogaster_AFH06455_gilgamesh_isoJ
D_melanogaster_NP_001247137_gilgamesh_isoJ
D_melanogaster_NP_001014628_gilgamesh_isoF
D_melanogaster_NP_001262624_gilgamesh_isoL
D_melanogaster_NP_788683_gilgamesh_isoE
Drosophila_virilis_XP_032289459
Drosophila_willistoni_XP_023035705
Ceratitis_capitata_XP_023158491
Ceratitis_capitata_XP_023158492
Ceratitis_capitata_XP_023158493
Ceratitis_capitata_XP_020718035
Ceratitis_capitata_XP_020718038
Ceratitis_capitata_XP_023158495
Ceratitis_capitata_XP_023158494
Ceratitis_capitata_XP_020718036
Musca_domestica_XP_019894430
Musca_domestica_XP_019894432
Musca_domestica_XP_019894431
Musca_domestica_XP_019894433
Anopheles_albimanus_XP_035782080
Anopheles_albimanus_XP_035782093
Anopheles_albimanus_XP_035782088
Anopheles_albimanus_XP_035782089
Anopheles_albimanus_XP_035782092
Anopheles_albimanus_XP_035782086
Anopheles_albimanus_XP_035782091
Culex_quinquefasciatus_XP_038116328
Culex_quinquefasciatus_XP_038116330
Culex_quinquefasciatus_XP_038116331
Mus_musculus_NP_001153063_CKIgamma-2_iso2
Mus_musculus_NP_775277_CKIgamma-1
Mus_musculus_EDL09901_CKIgamma3
Mus_musculus_EDL09899_CKIgamma3
Mus_musculus_EDL09900_CKIgamma3
2.0
